# The Marine-Derived Cyclopentapeptide Turnagainolide B Suppresses Melanoma via Autophagic Flux Disruption and Inhibits Tumorigenesis In Vivo

**DOI:** 10.3390/md24070235

**Published:** 2026-07-03

**Authors:** Guoyue Wan, Keyu Zhao, Min Wang, Ren-He Xu, Meiling Jin, Liwei Liu

**Affiliations:** 1 Center of Reproduction, Development & Aging, Cancer Center and Institute of Translational Medicine, Faculty of Health Sciences, University of Macau, Taipa, Macau 999078, China; yc27675@um.edu.mo (G.W.); renhexu@um.edu.mo (R.-H.X.); 2 State Key Laboratory of Quantitative Synthetic Biology, Shenzhen Institute of Synthetic Biology, Shenzhen Institutes of Advanced Technology, Chinese Academy of Sciences, Shenzhen 518000, China; 3 Department of Dermatology, The First Affiliated Hospital of Ningbo University, Ningbo 315010, China; 4 College of New Materials and Chemical Engineering, Beijing Institute of Petrochemical Technology, Beijing 102617, China; 5 Li Dak Sum Yip Yio Chin Kenneth Li Marine Biopharmaceutical Research Center, Health Science Center, Ningbo University, Ningbo 315211, China

**Keywords:** marine natural product, turnagainolide B, melanoma, autophagy, bioactive, tumorigenesis

## Abstract

Melanoma remains highly lethal with frequent resistance to current therapies. Here we identify a marine-derived cyclopentapeptide, turnagainolide B, as a potent anti-melanoma agent that selectively kills B16-F10 melanoma cells (IC_50_ = 50 μM) with low toxicity to normal skin cells. Using bioassay-guided isolation, we also obtained a new analogue, turnagainolide H, and elucidated their structures and biosynthetic pathways. Mechanistically, turnagainolide B induces a previously undescribed “dual-hit” autophagic signature: it simultaneously promotes autophagy initiation (via PI3K/mTOR suppression, evidenced by ATG5 and LC3B-II upregulation) and blocks autophagic degradation (evidenced by p62 accumulation). Co-treatment with chloroquine partially rescued cell viability and decreased LC3B levels, confirming that cell death depends on active autophagic flux disruption. Transcriptomic analysis, together with AI target prediction and docking, identified PI3K as a potential direct target, with downregulation of PI3K, mTOR, and BNIP3 supporting an imbalanced autophagic state. In a syngeneic mouse melanoma model, turnagainolide B significantly suppressed tumor growth, reduced melanin content and Ki67 expression, and enhanced CD8^+^ T cell infiltration. Collectively, this work expands the chemical diversity of the turnagainolide family, uncovers a unique “dual-hit” autophagic mechanism, and establishes turnagainolide B as a promising lead for melanoma therapy.

## 1. Introduction

Melanoma is the deadliest form of skin cancer. Although early-stage melanoma is curable by surgical resection, the prognosis for metastatic disease remains dismal, with a 5-year survival rate below 30% [1,2]. Despite advances in targeted therapy and immunotherapy, primary and acquired resistance inevitably develop in a substantial proportion of patients [3,4,5,6]. This therapeutic bottleneck underscores an urgent, unmet need for new drugs with entirely different mechanisms of action.

Current therapeutic strategies predominantly rely on inducing apoptosis. However, tumor cells frequently evade this fate by activating pro-survival pathways, most notably autophagy [7,8]. Autophagy is a conserved lysosomal degradation pathway that maintains cellular homeostasis [9]. Interestingly, autophagy plays a double-edged sword in cancer: it helps cells cope with stress, but when excessively or aberrantly activated, it can trigger autophagic cell death [10,11,12]. This duality positions autophagy as an attractive therapeutic target. Rather than simply inhibiting or activating autophagy, strategies aimed at disrupting autophagic equilibrium, inducing a state of “autophagic turmoil”, may create a unique vulnerability in tumor cells [13,14,15]. Lipidation of LC3B-I to LC3B-II marks autophagosome formation, while P62/SQSTM1, a receptor that delivers ubiquitinated cargo for degradation, is consumed during functional autophagic flux [16,17]. Their co-accumulation therefore serves as a hallmark of impaired autophagic degradation [16]. Persistent dysregulation of these processes can trigger lysosomal membrane permeabilization, metabolic catastrophe, and ultimately cell death that may bypass the resistance mechanisms limiting current melanoma therapies [18,19,20,21].

Naturally synthesized peptides possess various potential biological and therapeutic functions, especially for marine-derived cyclic peptides as promising lead compounds for targeting protein–protein interactions (PPIs), owing to their conformational rigidity and enhanced metabolic stability [22,23,24,25]. Accordingly, our group has long focused on the discovery and functional characterization of bioactive peptides from marine microorganisms, which represent a largely untapped reservoir of chemical diversity with considerable therapeutic potential [24]. Turnagainolide peptides were originally isolated from the marine bacterium *Bacillus* sp. *RJA2194*, which was obtained from a sediment core sample at 104 m depth in Howe Sound, British Columbia, Canada [26]. In our previous study [27], we reported the genome sequence of Bacillus subtilis LP and identified the tur biosynthetic gene cluster responsible for turnagainolide production. The present study builds on this foundation by isolating and characterizing additional turnagainolide analogues. Using bioassay-guided isolation, we obtained turnagainolide B, which has been previously reported as an activator of SHIP1 in enzymatic assays [26], and a new analogue, turnagainolide H. Their structures and biosynthetic pathways were then elucidated. Among these, preliminary screening revealed that turnagainolide B exhibits potent anti-proliferative activity against melanoma cells. This finding prompted us to systematically evaluate its anti-melanoma potential and, more importantly, to elucidate how it kills melanoma cells.

Here we demonstrate that turnagainolide B kills melanoma cells through a previously undescribed “dual-hit” autophagic mechanism, fundamentally distinct from classical autophagy modulators that either induce or block autophagy alone. Functionally, turnagainolide B inhibits melanoma cell proliferation and migration by inducing this unique autophagic signature: it promotes autophagosome formation via PI3K/mTOR suppression (evidenced by ATG5 and LC3B-II upregulation) while simultaneously blocking autophagic degradation (evidenced by p62 accumulation and chloroquine co-treatment studies). This “dual-hit” creates a state of autophagic flux dysregulation—neither simple induction nor simple blockade—leading to catastrophic accumulation of undegraded cargo and ultimately autophagic cell death. In vivo, turnagainolide B suppresses tumor growth, reduces melanin content and Ki67 expression, and enhances CD8^+^ T cell infiltration, with low toxicity toward normal skin cells.

Collectively, this work establishes turnagainolide B as a promising new lead for melanoma therapy, operating through a unique autophagic mechanism that bypasses conventional apoptosis pathways and may overcome resistance to existing treatments.

## 2. Results

### 2.1. New Anti-Melanoma Natural Products Screening and Identification

To identify lead compounds with novel anti-melanoma mechanisms, B16-F10 melanoma cells were employed as a screening model, and our in-house marine bacterial fermentation extract library was subjected to large-scale screening using an MTT-based cell viability assay. Among the tested samples, the fermentation extract of the marine-derived bacterium *B. subtilis* LP exhibited pronounced antitumor activity. Guided by bioassay-directed fractionation, one known compound, turnagainolide B (**1**), and one new congener, turnagainolide H (**2**), were subsequently identified.

Turnagainolide B (**1**) was confirmed to be identical to the previously reported data [27]. It displayed an [M+H]^+^ ion at *m*/*z* 557.3328 (calcd for C_30_H_45_O_6_N_4_, 557.3339; Δm = 2.0 ppm) and exhibited the same UV absorption spectrum (Figures S1 and S2). The structure was further confirmed by extensive NMR spectroscopic analysis (Figure S3 and Table 1).

Turnagainolide H (**2**) was obtained as a white powder and exhibited an [M+H]^+^ ion at *m*/*z* 543.3182 in the HRESIMS spectrum (calcd for C_29_H_43_O_6_N_4_, 543.3183; Δ = 0.2 ppm) (Figure S2). The molecular formula was established as C_29_H_42_O_6_N_4_, corresponding to 11 degrees of unsaturation. Analysis of the ^1^H and HSQC NMR spectra revealed the presence of four amide NH signals (δ_H_ 7.67, 7.87, 8.20, and 8.71), a monosubstituted benzene ring [δ_H_ 7.44 (H-7/H-11), 7.35 (H-8/H-10), and 7.28 (H-9)], and two olefinic protons (δ_H_ 6.27 and 6.67). The ^13^C, HSQC, and HMBC spectra further indicated the presence of five carbonyl carbons (δ_C_ 168.74, 169.06, 169.80, 170.46, and 172.66), along with resonances corresponding to a monosubstituted phenyl ring and two olefinic carbons [δ_C_ 126.60 (2C), 126.78, 128.26, 128.82 (2C), 132.54, and 135.79]. These features accounted for 10 of the 11 required degrees of unsaturation, suggesting that compound **2** possesses a monocyclic framework.

Detailed analysis of the COSY, HSQC, and HMBC data (Figure S4) showed that the ^13^C NMR signals at δ_C_ 135.79 (Hppa-1, C-6), 126.60 (Hppa-1, C-7/C-11), 128.82 (Hppa-1, C-8/C-10), and 128.26 (Hppa-1 C-9) correspond to a monosubstituted phenyl ring. Continuous COSY correlations from H-2 to H-5 (Hppa-1), together with the HMBC correlation from H-2 to C-1 (Hppa-1), supported the presence of a 3-hydroxy-4-pentenoic acid moiety. Furthermore, HMBC correlations from H-5 to C-6 and from H-7 to C-5 established the connection between the phenyl ring and the 3-hydroxy-4-pentenoic acid fragment, forming the Hppa unit. The large coupling constant (J = 16.0 Hz) between the two olefinic protons indicated an *E* configuration of the double bond.

**Figure 1:**
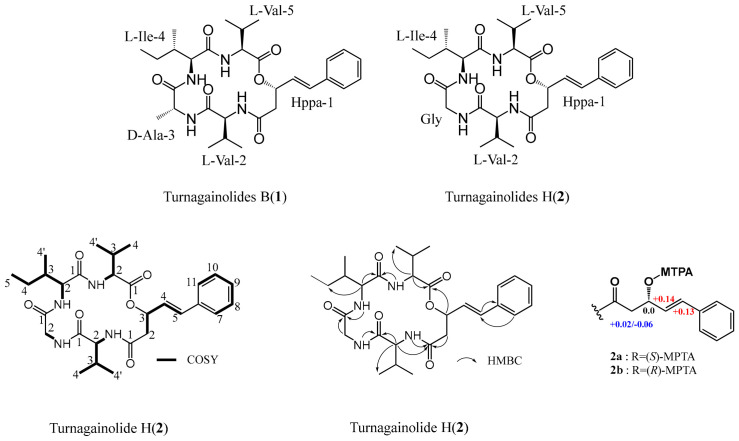
Structures of turnagainolides B (**1**), and H (**2**) with residue names; Key 2D NMR correlations of turnagainolide H (**2**) and Mosher ester analysis of the Hppa residue in **2**. Abbreviation: Hppa, 3-hydroxy-4-pentenoic acid; MTPA, α-methoxy-α-(trifluoromethyl)phenylacetic acid. (Mosher’s reagent).

Inspection of the ^1^H NMR and COSY spectra revealed four α-proton signals corresponding to amino acid residues. HMBC correlations allowed assignment of a Val-Gly-Ile-Val peptide sequence (Val-2, Gly-3, Ile-4, and Val-5). Key HMBC correlations from NH (Val-2) to C-1 (Hppa-1) and from H-3 (Hppa-1) to C-1 (Val-5) established that the Hppa unit is located between Val-2 and Val-5 and that the molecule is cyclized through ester and amide linkages, thereby satisfying the final degree of unsaturation (Figure S4). Comparison with turnagainolide B (**1**) revealed that compound **2** shares the same overall scaffold, except that the Ala-3 residue in **1** is replaced by Gly-3 in **2** (Table 1, Figure 1 and Figure S4). Accordingly, compound **2** was designated as turnagainolide H following the established nomenclature for this family of compounds.

To determine the stereochemistry of turnagainolide H (**2**), we first used Marfey’s analysis to establish the configurations of the amino acids. Our results showed that the amino acids in turnagainolide H (**2**) were predominantly L-configured (Figure S5). Subsequently, we utilized NaOMe to catalyze methanolysis of the ester linkage in turnagainolide H (**2**), exposing the hydroxyl group. Mosher ester analysis was then employed to establish the configuration at C-3 (Hppa-1) (Figure 1 and Figures S6 and S7). The configuration of C-3 in the Hppa residue was determined as *S* in turnagainolide H (**2**).

### 2.2. The Putative Biosynthetic Pathway of Turnagainolides B and H

**Figure 2:**
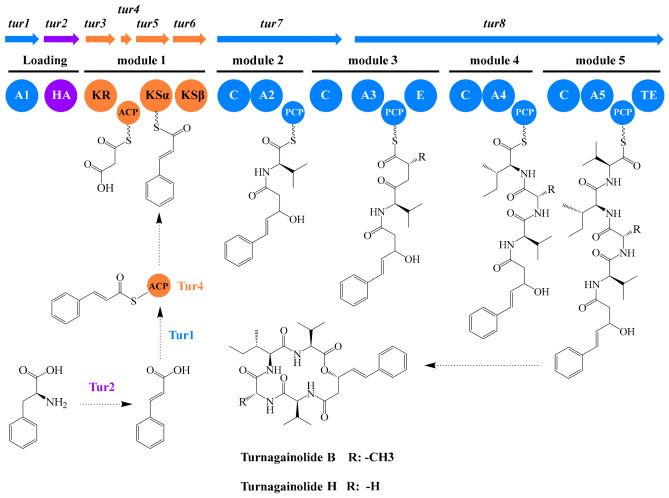
The putative biosynthetic pathway of turnagainolides B and H. Abbreviations: tur1–8, open reading frames within the tur biosynthetic gene cluster (BGC), responsible for turnagainolide biosynthesis; A, adenylation domain; ACP, acyl carrier protein in PKS; C, condensation domain; E, epimerase domain; HA, histidine ammonia-lyase domain; KSα, keto-synthase domain; KSβ, keto-synthase domain; KR, keto-reductase domain; PCP, peptidyl-carrier protein; TE, thioesterase domain.

Although we previously elucidated the overall biosynthetic logic of turnagainolides in *B. subtilis* LP [27], the identification of the new congener turnagainolide H (**2**) provides additional evidence for refining the biosynthetic pathway of this family. Accordingly, the pathway was re-evaluated based on the structure of turnagainolide H, antiSMASH analysis, and our previous in-depth investigations (Figure 2) [27].

Biosynthesis is proposed to initiate with the deamination of L-phenylalanine (L-Phe), followed by its conversion into the Hppa residue. Notably, the ketoreductase (KR) domain exhibits 71.78% sequence identity to FabG and is likely responsible for establishing the C-3 stereochemistry of Hppa, showing strong stereoselectivity toward the *S*-configuration during the biosynthesis of turnagainolides B and H. A key finding of this study is the functional reassignment of NRPS module 3 (Tur7). Previous studies suggested that this module incorporates D-Ala-3, consistent with the presence of an epimerization (E) domain in turnagainolide B. However, structural analysis of turnagainolide H revealed the incorporation of Gly-3 at the corresponding position. Further analysis of the A3 domain substrate-binding pocket (specificity-conferring code) suggested notable substrate promiscuity. In the biosynthesis of turnagainolide H, this module appears to bypass epimerization and directly incorporate Gly. Finally, macrocyclization is catalyzed by the thioesterase (TE) domain in Tur8, which remains highly conserved. The TE domain mediates macrolactone formation between the hydroxyl group of Hppa and the carbonyl group of L-Val-5 via the conserved “GXSXG” motif [28], yielding the 17-membered ring structure.

### 2.3. Preliminary Bioactivity Profiling of Turnagainolides B

#### 2.3.1. Turnagainolide B Can Effectively Inhibit Tumor Growth

**Figure 3:**
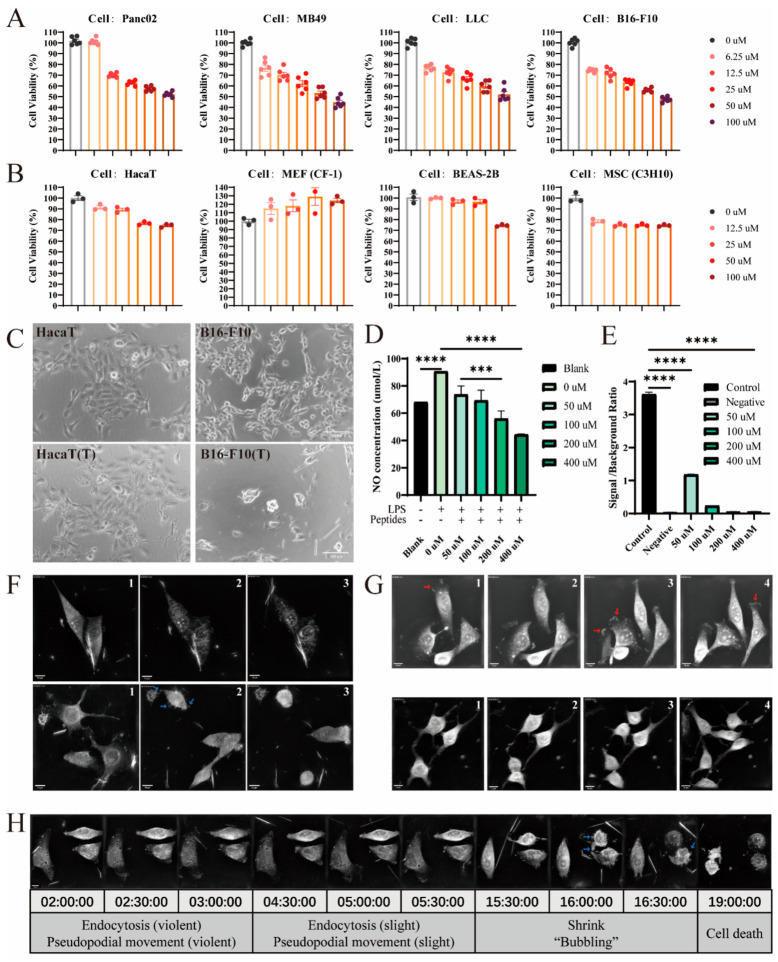
Preliminary bioactivity profiling of turnagainolide B. (**A**) MTT test of turnagainolide B for four cancer cells. (**B**) MTT test of turnagainolide for four normal cells. *p* < 0.001 for all comparisons in (**A**,**B**). (**C**) Comparison photos of cells before and after drug administration (optical microscope). Scale bar = 100 µm. (**D**) Turnagainolide B inhibits LPS-induced NO production in BV2 microglial cells. Cells were pretreated with the indicated concentrations of turnagainolide B (0, 50, 100, 200, 400 µM) for 1 h, then stimulated with LPS (1 µg/mL) for 24 h. NO levels in the culture supernatant were measured using the Griess assay. Data are presented as mean ± SEM (*n* = 3). (**E**) HDAC inhibitory activity of turnagainolide B in MB49 cells. MB49 cells were treated with the indicated concentrations of turnagainolide B (0, 50, 100, 200, 400 µM) for 24 h. HDAC activity was measured using a fluorometric HDAC activity assay kit. Data are presented as mean ± SEM (*n* = 3). (**F**,**H**) live cell image of B16-F10 with 50 μM turnagainolide B in culture medium for 24 h. Cell rupture (top) or shrinkage (bottom) leads to death. Blue arrow: Bubble. Scale bar = 10 µm. (**G**) live cell image of B16-F10 without treatment. Red arrow: pseudopodia. Scale bar = 10 µm. Number highlights the distinct phases in time sequence of cell death leading to lysis in (**F**,**G**) (see Supplementary_video for the complete dynamics). (*** *p*  <  0.001, **** *p*  <  0.0001).

To identify an appropriate model for mechanistic study, we selected turnagainolide B as the major target against further four tumor cell lines, because of its good performance and higher yield from fermentation (Figure S8) [27]. Due to the extremely low yield of turnagainolide H, we were unable to perform a full dose–response curve to confirm its IC_50_; however, preliminary experiments clearly demonstrated that turnagainolide H exhibited enhanced anti-proliferative activity against two mouse tumor cells lines compared to turnagainolide B (Figure S8). Therefore, we chose B for the following experiment. In this content, turnagainolide B demonstrated robust, dose-dependent cytotoxicity across multiple cancer cell lines, with pronounced activity against B16-F10 melanoma cells (Figure 3A). Given its potent anti-melanoma activity and efficient production, turnagainolide B and B16-F10 cells were chosen for mechanistic studies. Flow cytometry results further show that turnagainolide B induces apoptosis in B16-F10 cells at a concentration of 50 μM (Figure S9). Importantly, turnagainolide B exhibited significantly lower cytotoxicity toward normal skin-associated cells, including epidermal keratinocytes (HaCaT), mouse embryonic fibroblasts (MEF, CF1), bronchial epithelial cells (BEAS-2B), and mesenchymal stem cells (MSC, C3H10), suggesting a favorable safety profile for potential local application (Figure 3B,C). Phase-contrast microscopy further corroborated this differential response: after 24 h treatment with 50 μM turnagainolide B, B16-F10 cells displayed marked morphological changes, including cytoplasmic vacuolization, detachment, and cell death, whereas HaCaT cells remained largely unaffected (Figure 3C and Figure S10).

#### 2.3.2. Turnagainolide B Possesses HDAC Inhibitory and Anti-Inflammatory Activity

To further investigate the mechanism underlying turnagainolide B-induced melanoma cell death, we next investigated whether turnagainolide B exhibits additional activities relevant to its anti-cancer potential. Meanwhile, given that HDAC inhibition represents a classic mechanism of action for several clinically approved anticancer agents [29], we assessed its HDAC inhibitory activity alongside its anti-inflammatory effects. In LPS-stimulated BV2 microglial cells, higher concentrations of turnagainolide B effectively suppressed the LPS-induced production of nitric oxide (NO), demonstrating its anti-inflammatory effect (Figure 3D). Furthermore, in MB49 cells, turnagainolide B at 50 μM markedly inhibited HDAC activity, compared to a known HDAC inhibitor valproic acid (VPA) (Figure 3E). These findings reveal that turnagainolide B possesses both anti-inflammatory and HDAC inhibitory activities, further supporting its therapeutic potential.

#### 2.3.3. Label-Free Live Cell Imaging Reveals a Unique Temporal Cascade of Cell Death Induced by Turnagainolide B Related to Autophagy

To dynamically capture the cytotoxic effects of turnagainolide B, we performed label-free, continuous Nanolive holotomographic microscopy over 24 h. Treatment with 50 µM turnagainolide B triggered a stereotyped sequence of morphological events culminating in cell death. Cells progressively lost motility and pseudopodial activity, followed by two distinct death trajectories (Figure 3F). One subset remained relatively static for up to 18 h, exhibiting intense membrane blebbing that culminated in explosive lysis, leaving shadow-like remnants (upper panel). Another population underwent rapid progressive shrinkage within 12 h to 14 h, accompanied by violent cytoplasmic “boiling” and the formation of apoptotic-like vesicles prior to death (lower panel). In stark contrast, untreated control B16-F10 cells displayed active motility, dynamic pseudopodial extensions, and normal cell division throughout the imaging period (Figure 3G, Supplementary_video). This real-time visualization provides direct evidence that turnagainolide B induces a slow, inexorable, and morphologically distinct form of cell death, one that is clearly distinguishable from rapid necrotic lysis (Figure 3H).

### 2.4. Turnagainolide B Downregulates PI3K/mTOR and BNIP3

#### 2.4.1. mRNA Profile Analysis Reveals Downregulation of PI3K/mTOR and BNIP3, Prompting Functional Validation of Autophagic Flux and Cell Migration

**Figure 4:**
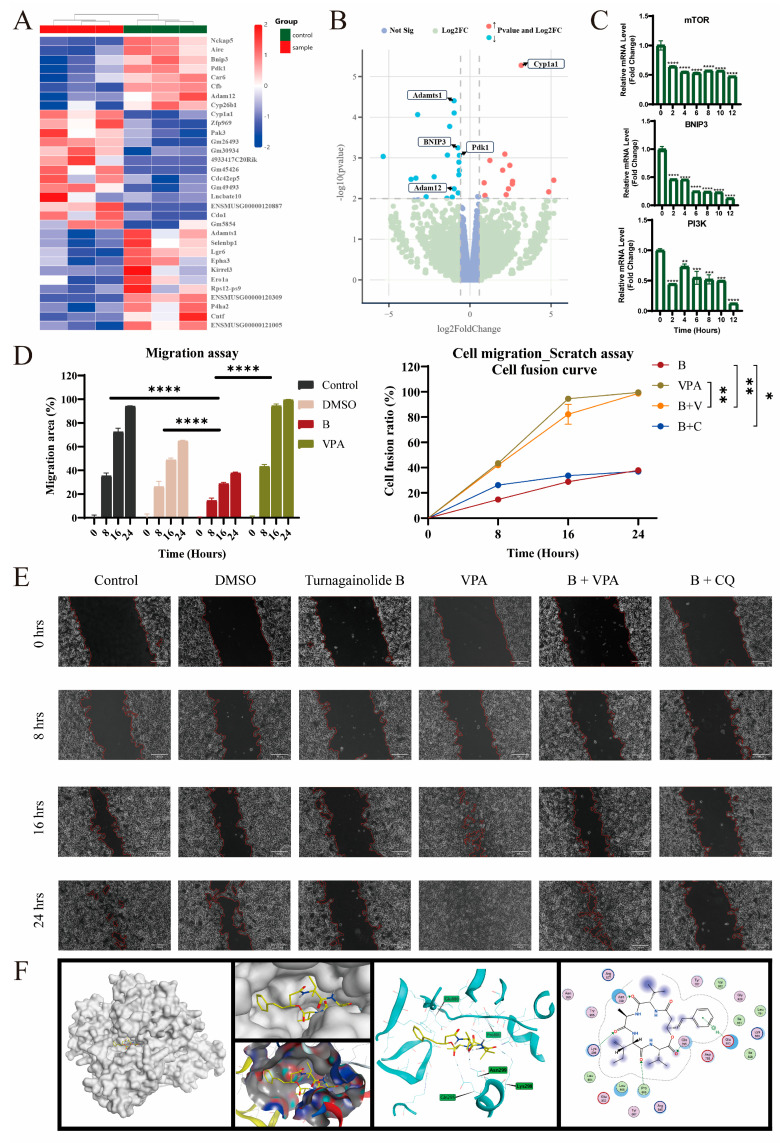
mRNA profile analysis reveals a perturbed transcriptome related to cellular motility and death in turnagainolide B-treated melanoma cells. (**A**) Cluster analysis of differentially expressed genes. Red represents up-regulation, blue represents down-regulation, and the intensity of color indicates the degree of up- or down-regulation. (**B**) Volcano plot analyzed fold change of three independent batch of cells. (**C**) qPCR result of mTOR, PI3K and BNIP3 in first 12 h after incubating with turnagainolide B. (**D**) Quantification of cell migration area and fusion ratio. Fusion ratio was calculated as [1 − (wound area at 24 h/wound area at 0 h)] × 100%. Data are presented as mean ± SEM (*n* = 6). Abbreviations: B, turnagainolide B; V, valproic acid; C, chloroquine. (**E**) Representative images of the migration assay. B16-F10 cells were scratched and then treated with vehicle control (0.1% DMSO), 50 µM turnagainolide B, 3.3 mM valproic acid (VPA), 20 µM chloroquine (CQ), or combinations as indicated. Images were taken at 0, 8, 16 and 24 h post-scratch. Scale bar = 200 µm. (**F**) Molecular docking of turnagainolide B with the PI3Kγ ATP-binding pocket (PDB: 3L54). Key interacting residues are labeled: Lys298, Asn299, Gln295, Pro866 (hydrogen bonds, blue dashed lines) and Glu880 (arene-H interaction, green dashed line). (* *p*  <  0.05, ** *p*  <  0.01, *** *p*  <  0.001, **** *p*  <  0.0001).

To gain genome-wide insights into the transcriptional changes induced by turnagainolide B, we performed RNA-seq on B16-F10 cells treated with 50 μM turnagainolide B or vehicle control with three rounds of independently repeated experiments. Among 56,749 expressed transcripts, 32 were identified as differentially expressed genes (DEGs) (fold change ≥ 1.5, *p* < 0.01) upon treatment (Figure 4A,B and Figure S11). Notably, key components of the PI3K/mTOR signaling axis and autophagy pathway, including PI3K, mTOR, and BNIP3, were significantly downregulated, which was further validated by qPCR (Figure 4C). BNIP3, a mitochondrial receptor, further contributes to mitochondrial homeostasis by mediating mitophagy [30]. In parallel, in silico target prediction identified Pik3ca-p110, a key catalytic subunit of PI3K, as a potential target, consistent with the qPCR results (Supporting Table). It is worth noting that suppression of the PI3K/mTOR axis promotes early-stage autophagosome formation, while downregulation of BNIP3 may impair late-stage cargo sequestration, collectively creating an imbalanced autophagic flux. Additionally, several migration-related genes, such as Adamts1, Adam12, and Cdc42ep5, were also markedly suppressed (Figure 4A and Figure S11) [31,32,33].

To functionally validate these findings, we performed a wound healing assay. Turnagainolide B treatment significantly impaired B16-F10 cell migration, as evidenced by a markedly slower fusion rate compared to controls (Figure 4D,E). Intriguingly, this anti-migratory effect was substantially reversed by co-treatment with the HDAC inhibitor VPA, but not by the autophagy inhibitor chloroquine (CQ) (Figure 4E). Notably, VPA alone induced marked cellular hypertrophy (see below), a morphological change that should be considered when interpreting the recovered migration speed in the combination group, as it may reflect factors beyond motility itself.

Together, these transcriptomic data reveal that turnagainolide B simultaneously modulates autophagy initiation (via PI3K/mTOR suppression) and late-stage cargo recruitment (via BNIP3 downregulation), creating an imbalanced autophagic state that may underlie its anti-melanoma activity. To functionally validate this autophagic perturbation, we next examined key markers of autophagic flux.

#### 2.4.2. AI Target Prediction and Docking Suggest Direct PI3Kγ Binding of Turnagainolide B

Given that turnagainolide B transcriptionally downregulated PI3K (Figure 4C), we next investigated whether it can directly interact with PI3K. In silico target prediction using SwissTargetPrediction identified PIK3CG-p110, a key catalytic subunit of class I PI3K, as a potential target (Supporting Table). To further validate this, we performed molecular docking using the PI3Kγ crystal structure (PDB: 3L54), with a higher binding energy: −8.03 kcal/mol, the binding pocket of which is highly conserved among class I PI3K isoforms. Compared to the previous research of PI3K inhibitor in 3L54, turnagainolide has predominant key interactions, including hydrogen bonds with Lys298, Asn299, Gln295, and Pro866, as well as an arene-H interaction with Glu880 (Figure 4F). These in silico results provide a structural basis for the transcriptional downregulation of PI3K observed upon turnagainolide B treatment (Figure 4C), supporting a direct targeting mechanism.

### 2.5. Turnagainolide B Induces a Time-Dependent, Dual-Hit Dysregulation of Autophagic Flux

**Figure 5:**
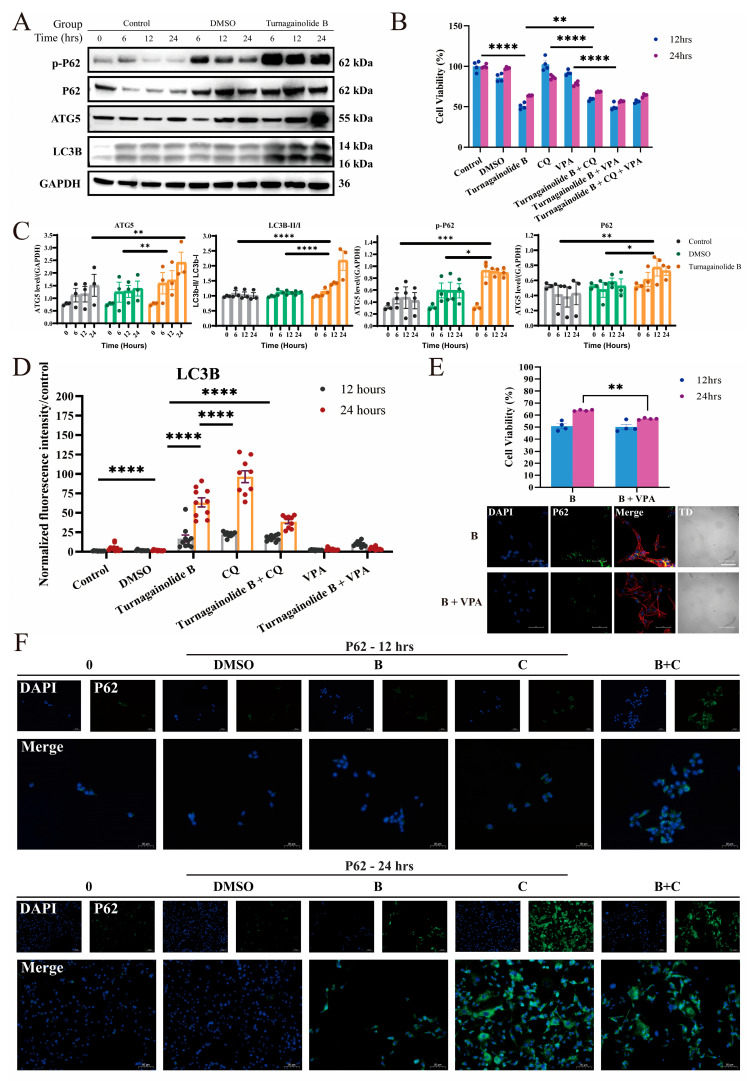
Turnagainolide B induces a time-dependent dysregulation of autophagic flux. (**A**,**C**) Western blot analysis of LC3B-II, p62, and ATG5 in B16-F10 cells treated with 50 µM turnagainolide B for 24 h, and its quantitative analysis. (**B**) MTT cell viability assay under the indicated conditions. Abbreviations: B, turnagainolide B; C, chloroquine. (**D**) Quantitative analysis of immunofluorescence staining for anti-LC3B. (**E**) p62 immunofluorescence at 24 h for the turnagainolide B alone and turnagainolide B + VPA groups. Abbreviations: DAPI, 4′,6-diamidino-2-phenylindole for DNA staining; TD, Transmitted Detector. Red: Actin-Tracker of cell structure. (**F**) p62 immunofluorescence at 12 h and 24 h. The key difference between (**E**) and (**F**) is that (**E**) focuses on the effect of VPA co-treatment at 24 h, while (**F**) shows the time-dependent accumulation of p62 from 12 h to 24 h. (* *p*  <  0.05, ** *p*  <  0.01, *** *p*  <  0.001, **** *p*  <  0.0001). Scale bar = 50 µm (applies to (**E**,**F**)).

To elucidate the molecular mechanism underlying its bioactivity, we examined key autophagy markers by Western blot. In B16-F10 cells treated with 50 μM turnagainolide B for 24 h, we observed a concurrent accumulation of LC3B-II and P62, a classic hallmark of impaired autophagic flux (Figure 5A,C). ATG5 expression was simultaneously upregulated (Figure 5A,C), indicating active autophagosome formation, whereas P62 accumulation pointed to a blockade at later stages of autophagy. These changes were accompanied by downregulation of BNIP3, mTOR, and PI3K (Figure 4C), further supporting multi-level dysregulation of autophagy signaling by turnagainolide B.

Immunofluorescence analysis was next assessed to dissect the autophagic response. After 12 h of treatment, turnagainolide B induced a modest increase in LC3B puncta formation relative to controls, indicating enhanced autophagosome biogenesis (Figure 5D). Co-treatment with CQ attenuated this LC3B signal, whereas co-treatment with VPA enhanced it (Figure 5D). Unlike turnagainolide B, VPA alone did not increase LC3B puncta or P62 levels at 12 or 24 h; however, adding VPA to turnagainolide B significantly reduced P62 puncta at 24 h (Figure 5E). Notably, VPA-treated cells exhibited marked cellular hypertrophy (Figure 5E). However, at this early time point, P62 fluorescence intensity remained unchanged across groups (Figure 5F). This pattern shifted dramatically by 24 h, P62 accumulated robustly in turnagainolide B-treated cells, demonstrating a hallmark co-accumulation indicative of autophagic flux blockade (Figure 5F). Interestingly, combining turnagainolide B with CQ did not further enhance the fluorescence signal, though cell number increased modestly compared to B alone group (Figure 5F).

### 2.6. Turnagainolide B Induces Autophagic Cell Death: Evidence from Chloroquine Rescue

A critical distinction must be made between “cell death with autophagy” and “autophagic cell death” [14]. The former describes scenarios where autophagy accompanies but does not execute cell death, often representing a failed survival attempt. The latter, by contrast, refers to cell death that is mechanistically dependent on the autophagic machinery itself, typically characterized by massive cytoplasmic vacuolization, persistent autophagic flux blockade, and non-apoptotic execution [34]. To distinguish between these possibilities, we utilized key pharmacological modulators of autophagy: CQ, which inhibits autophagic flux by alkalinizing lysosomes, and VPA, an HDAC inhibitor known to modulate epigenetic states and cell migration [35,36]. Strikingly, co-treatment with CQ partially rescued cell viability compared to turnagainolide B alone (Figure 5B), accompanied by a decrease in LC3B levels (Figure 5D). No additive effect was observed when turnagainolide B was combined with CQ. In contrast, VPA co-treatment did not alter cell viability. Notably, among all groups, turnagainolide B alone exhibited the weakest cell viability (Figure 5B). These results demonstrate that turnagainolide B-induced autophagy directly mediates cytotoxic effects, confirming that turnagainolide B induces autophagic cell death rather than cell death with autophagy.

### 2.7. Turnagainolide B Suppresses Melanoma Growth and Modulates Anti-Tumor Immunity In Vivo

**Figure 6:**
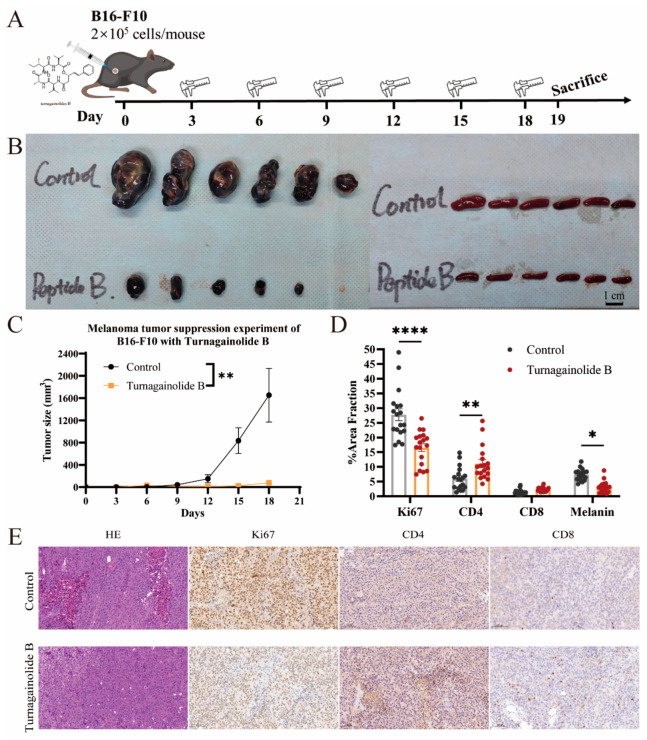
Animal experiment of turnagainolide B. (**A**) Experimental timeline for the syngeneic C57BL/6J mouse model. (**B**) Representative photographs of excised tumors (**right**) and spleens (**left**) from vehicle control and turnagainolide B-treated mice (written as peptide B on this photo). (Bar = 1 cm). (**C**) Tumor growth curves over 18 days. (**D**) Bar graphs showing quantitative analysis of quantification of Ki67-positive cells, CD4^+^/CD8^+^ T cell infiltration and melanin content of solid tumors. (**E**) Representative histopathological images of tumor tissues: HE staining (showing melanin content), Ki67 immunohistochemistry (proliferation marker), CD4 and CD8 immunohistochemistry (T cell infiltration) of solid tumors. (Bar = 100 um). (* *p*  <  0.05, ** *p*  <  0.01, **** *p*  <  0.0001).

Having established that turnagainolide B induces autophagic cell death in vitro, we next evaluated its therapeutic potential in vivo. C57BL/6J mice were subcutaneously implanted with 2 × 10^5^ B16-F10 melanoma cells and monitored for 18 days following treatment (Figure 6A and Figure S13). Turnagainolide B treatment significantly suppressed tumor growth compared to vehicle control, with treated mice exhibiting markedly smaller tumors and normal spleens (Figure 6B–D and Figure S13). Macroscopically, tumors from the treatment groups appeared less pigmented (whitish) at early stages (Figure 6B). Histopathological examination by H&E staining confirmed a reduction in melanin content within the tumors (Figure 6D,E). Immunohistochemical staining further revealed a decrease in the proliferation marker Ki67 upon turnagainolide B treatment (Figure 6D,E). Notably, tumors from treated mice displayed increased infiltration of CD8^+^ T and CD4^+^ T cells (Figure 6D,E), suggesting that turnagainolide B may not only directly inhibit tumor growth but also modulate the anti-tumor immune response. Collectively, these in vivo findings corroborate the in vitro mechanism and highlight the potential of turnagainolide B for topical melanoma therapy.

## 3. Discussion

Marine bacteria-derived natural products have emerged as a valuable resource for the discovery of anti-cancer drug leads [37]. Here, we identified the known cyclic peptide turnagainolide B and a new analogue, turnagainolide H, from *B. subtilis* LP with anti-melanoma activity. By integrating chemical isolation, structural elucidation, biosynthetic analysis, and systematic pharmacological evaluation [27], we demonstrate that turnagainolide B exerts anti-tumor activity through a distinctive mechanism: it simultaneously promotes autophagy initiation while blocking autophagic degradation, creating a state of autophagic flux dysregulation distinct from that of classical autophagy inhibitors [19,35,38,39].

To further explore structure-activity relationships, we compared turnagainolide B with its newly identified analogue H. Structural analysis revealed that turnagainolide H differs from turnagainolide B only by the substitution of L-Ala-3 with Gly-3 (Figure 1), while owing to extremely low fermentation yield of turnagainolide H, further mechanistic investigation was not feasible. Notably, turnagainolide H exhibited enhanced anti-cancer activity compared to turnagainolide B, suggesting that incorporation of Gly-3 may improve the bioactivity of this scaffold, possibly by reducing steric hindrance or modulating hydrophobic interactions with the target protein.

In contrast, the previously reported analogues turnagainolides D and E, despite sharing highly similar structures with turnagainolides B and H (Figure S14), exhibited no detectable anticancer activity in this study. The key structural variation, namely substitution of L-Val-4 with L-Ile-4, may impair productive interactions with the target protein. Furthermore, turnagainolides A and F (Figure S14), which contain a 3*R*-Hppa residue, were inactive, underscoring the critical role of the *S*-configuration of Hppa in maintaining anti-cancer activity. Altogether, comparative analysis of turnagainolide analogues reveals that the *S*-configuration of Hppa is essential for activity, and subtle substitutions at residues 3 (Gly vs. Ala) and 4 (Ile vs. Val) can profoundly influence anticancer potency, providing critical structure–activity relationship insights for future optimization.

Compared with our previous study [27], the updated biosynthetic model suggests that the structural diversity of turnagainolides primarily arises from the substrate promiscuity of adenylation domains, which govern amino acid incorporation in the NRPS assembly line. In addition, the ketoreductase (KR) domain likely mediates the formation of the 3*R/S*-Hppa unit. Given that the limited yield of natural products remains a major bottleneck for pharmaceutical development [40], these findings not only deepen our understanding of turnagainolide biosynthesis but also provide a foundation for future strain engineering to improve their production.

The mechanistic core of our findings lies in the unique autophagic signature induced by turnagainolide B. We observed concurrent elevation of LC3B-II, P62, and ATG5, a triad that simultaneously indicates promoted autophagosome formation (LC3B-II and ATG5 upregulation) and impaired autophagic degradation (P62 accumulation) [16,41]. This co-accumulation is a definitive hallmark of blocked autophagic flux, most likely at the stage of autophagosome-lysosome fusion or lysosomal function [7,10,35,38,39]. Moreover, the biochemical results further corroborate the autophagic-like cell death characteristics previously observed in the nano-live live-cell imaging assays. RNA-seq and qPCR analyses further substantiate this mechanism, revealing broad transcriptional downregulation of key nodes within the PI3K/mTOR signaling axis and the mitophagy receptor BNIP3 [30,42,43,44]. To probe the upstream regulator responsible for these transcriptional changes, we performed AI-based target prediction and molecular docking. These computational approaches swiftly identified PI3K as a high-confidence candidate, which was subsequently validated by qPCR and Western blot analyses. Thus, the integration of AI prediction with experimental validation accelerated the mechanistic dissection of turnagainolide B. Notably, the downregulation of PI3K and mTOR relieves the brake on early-stage autophagy initiation [45,46,47], promoting phagophore formation [44]; conversely, BNIP3 downregulation may compromise late-stage cargo sequestration. This biphasic regulation, concurrently driving autophagosome biogenesis and impairing cargo clearance, provides an imbalanced autophagic state that underpins the observed anti-melanoma activity [20,41]. Interestingly, turnagainolide B has been reported as a SHIP1 agonist [26], which is known to regulate phosphoinositide metabolism critical for endomembrane homeostasis [47,48,49]. Although this study does not directly experimentally confirm this hypothesis, it may represent one potential explanation for how Turnagainolide B contributes to autophagic dysregulation for further research.

This mode of autophagic dysregulation is fundamentally distinct from classical autophagy inhibitors such as CQ, which passively enters and alkalinizes lysosomes, thereby blocking autophagic degradation [35]. Critically, the decrease in LC3B levels upon CQ co-treatment provides direct evidence of a negative feedback mechanism: when autophagic degradation is completely blocked, cells sense autophagosome accumulation and actively downregulate biogenesis, partially alleviating the “traffic jam” and contributing to the observed recovery in cell viability [34,50]. The absence of an additive effect with CQ, coupled with this partial rescue, confirms that turnagainolide B induces a unique form of autophagic cell death, one mechanistically dependent on active autophagic flux disruption, distinct from the simple lysosomal blockade of classical autophagy inhibitors [20].

The potent activity of turnagainolide B in melanoma is particularly noteworthy given the unique biology of melanocytic cells. Melanoma cells possess a highly active endosomal-lysosomal system dedicated to melanosome biogenesis and maturation [51,52], a feature that may render them especially vulnerable to perturbations in autophagic flux [53]. By disrupting endolysosomal membrane homeostasis, turnagainolide B interferes with autophagolysosome maturation, acidification, and ultimately autophagic flux [48], thereby exploiting a melanoma-specific vulnerability.

Beyond its direct cytotoxic effects, turnagainolide B exhibits a favorable safety profile toward normal skin cells and demonstrates robust in vivo efficacy, highlighting its potential for topical or locoregional therapy of cutaneous melanoma. Treatment significantly suppressed tumor growth, reduced melanin content [54], and Ki67 proliferation index [55], and enhanced CD8^+^ T cell infiltration [56]. A limitation of current study is the low yield of turnagainolide B, future in vivo studies may consider including positive control agents such as chloroquine to further validate the autophagy-dependent anti-melanoma mechanisms. These findings, combined with its unique “dual-hit” mechanism, position turnagainolide B as a promising lead for melanoma therapy, particularly for patients who have developed resistance to conventional apoptosis-based treatments [22].

## 4. Materials and Methods

### 4.1. Turnagainolide B Fermentation and Purification

#### 4.1.1. Fermentation

*B. subtilis* LP was isolated as mentioned in a previous publication [27]. Then, it was initially inoculated into two 1 L Erlenmeyer flasks, each containing 400 mL of R2A liquid seed medium (100%, Hopebio, Qingdao, China), and cultured at 28 °C for 2 days with shaking at 160 rpm. The seed culture (1%, *v*/*v*) was subsequently transferred into 125 × 1 L Erlenmeyer flasks, each containing 400 mL (total as 50 L) of R2A medium supplemented with 2% (*w*/*v*) macroporous adsorbent resin HP-20 (Solarbio, Beijing, China), 4 mL seed per flask, total 500 mL seed used. Large-scale fermentation was carried out at 28 °C for 7 days with shaking at 160 rpm.

#### 4.1.2. Compound Isolation and Purification

After fermentation, the HP-20 resin was collected by filtration, washed with distilled water, and air-dried for 48 h. The resin was then exhaustively extracted with 100% methanol until no further compounds were detected. The combined methanol extracts were concentrated under reduced pressure to yield 13 g of crude extract. The crude extract was subjected to silica gel column chromatography (300–400 mesh; column size: 50 × 4 cm) using a stepwise gradient of petroleum ether (PE)/ethyl acetate (EtOAc)/methanol (MeOH): PE (100%), PE/EtOAc (1:1), EtOAc (100%), EtOAc/MeOH (9:1, 8:2, 7:3, 1:1, 3:7), and MeOH (100%). Each solvent system was eluted three times. A total of 17 fractions were collected and analyzed by HPLC. Analytical HPLC was performed using a Luna C18 column (100 × 3 mm, 2.6 μm, Phenomenex, Torrance, CA, USA) with an injection volume of 50 μL. Elution was carried out using a gradient from 10% to 100% aqueous acetonitrile (MeCN) over 30 min at 25 °C, followed by washing with 100% MeCN between runs. Fractions eluted with EtOAc/MeOH (9:1) containing turnagainolides were combined, concentrated, and subjected to semi-preparative HPLC purification. Separation was performed on a Luna C18 column (250 × 10 mm, 5 μm, Phenomenex, Torrance, CA, USA) at a flow rate of 2 mL/min, using isocratic elution with 40% aqueous MeCN for 120 min at 25 °C. The collected fractions were combined and evaporated to yield purified compounds: turnagainolide B (17.1 mg, tR = 103.48 min), turnagainolide H (0.6 mg, tR = 81.02 min) from 50 L fermentation broth. All solvents used were of HPLC grade (Fisher Scientific, Waltham, MA, USA).

#### 4.1.3. Marfey’s Derivatization

Each compound (0.2 mg) was hydrolyzed in 0.5 mL of 6 M HCl at 110 °C for 19 h. After cooling to room temperature, the solvent was removed under reduced pressure, and the residue was reconstituted in 100 μL of water. To this solution, 100 μL of 1% L-FDLA (N-(α)-(5-fluoro-2,4-dinitrophenyl)-L-leucinamide (ABCR GmbH, Karlsruhe, Germany) in acetone and 20 μL of 1 M NaHCO_3_ were added. The reaction mixture was incubated at 37 °C for 1 h and subsequently quenched with 20 μL of 1 M HCl. Standard amino acids were derivatized under identical conditions. The derivatized samples were analyzed by LC-MS (Q Exactive, Thermo Scientific, Waltham, MA, USA) using a C18 column (Hypersil GOLD, 100 × 2.1 mm, 3 μm). Separation was achieved with a gradient of 20–80% aqueous MeCN over 40 min at a flow rate of 0.3 mL/min. For isoleucine analysis, a chiral column (Lux 3 μm i-cellulose-5, 150 × 3.0 mm) was employed to resolve L-Ile and L-allo-Ile, using a gradient of 20–60% MeCN over 90 min at a flow rate of 0.4 mL/min.

#### 4.1.4. Esterlysis of Compounds

The compound (1.5 mg) was dissolved in 2 mL of 5% NaOMe in MeOH and stirred at room temperature for 20 min. The reaction mixture was neutralized with 2 mL of 1 M HCl and extracted three times with an equal volume of ethyl acetate. The combined organic phases were evaporated under reduced pressure, and the residue was re-dissolved in methanol and purified by semi-preparative reversed-phase HPLC using isocratic elution with 30% aqueous MeCN.

#### 4.1.5. Mosher Derivatization

The compound (0.5 mg) was dissolved in 100 μL of pyridine, followed by the addition of 2 μL of (R)-MTPA-Cl. The reaction mixture was stirred at room temperature for 10 h and purified by semi-preparative reversed-phase HPLC using a C18 column (150 × 4.6 mm, 4 μm, Agilent, Santa Clara, CA, USA) with isocratic elution in 33% aqueous MeCN. The same procedure was performed using (S)-MTPA-Cl. The absolute configuration was determined by comparison of NMR data (Bruker Avance IIIHD 600 MHz, CDCl_3_) of the corresponding Mosher esters.

#### 4.1.6. Genome Sequencing and Anti-SMASH Prediction

Genomic sequence data (GenBank accession no. CP152362) was downloaded and analyzed using the default parameters of the web-based anti-SMASH 8.0 software [57].

### 4.2. Cell Culture

The B16-F10 melanoma cell line (CRL-6475, ATCC, Manassas, VA, USA), MB49 bladder cancer cell line (SCC148, Sigma–Aldrich, St. Louis, MO, USA), LLC Lewis Lung Carcinoma (CRL-1642, ATCC, USA), Panc02 (STCC20054P, ServiceBio, Wuhan, China), MEF (CF-1, SCRC-1040.1, ATCC, USA) BEAS-2B epithelial cell (CRL-3588, ATCC, Manassas, VA, USA), MSC (C3H10, CCL-226, ATCC, USA) were routinely cultured in Dulbecco’s Modified Eagle Medium (C11995500BT, Gibco, Waltham, MA, USA) supplemented with 10% fetal bovine serum (10099141C, Gibco, USA) and 1% Penicillin-Streptomycin (100X) (15140122, Gibco, USA) at 37 °C in a humidified incubator with 5% CO_2_. Cells were passaged every 2–3 days upon reaching 80–90% confluence using 0.25% trypsin-EDTA (25200072, Gibco, USA) for detachment and passage. For all experiments, cells in the logarithmic growth phase were used.

### 4.3. Cell Viability and Proliferation Assays

Cell viability was assessed using the MTT (Methylthiazolyldiphenyl-tetrazolium bromide, MTT, 40201ES72, Yeasen, Shanghai, China) assay. Briefly, cells were seeded in 96-well plates at a density of 5 × 10^3^ cells per well with 100 μL medium. After 24 h of attachment, cells were treated with varying concentrations of turnagainolide B (0, 12.5, 25, 50, 100 μM) or vehicle control (0.1% DMSO) for 24 h. For further testing, 20 μM CQ, 3.3 mM VPA was utilized for incubation. At each designated time point, 10 μL of MTT solution (5 mg/mL in PBS) was added to each well, followed by incubation at 37 °C for 4 h. Subsequently, the culture medium containing MTT was carefully aspirated, and 100 μL of dimethyl sulfoxide (DMSO) was added to each well to dissolve the formed formazan crystals. The absorbance of the solution was measured at a wavelength of 550–570 nm using a microplate reader, Agilent BioTek Synergy H1 Microplate Reader, (Agilent Technologies, Santa Clara, CA, USA). Cell viability was calculated as a percentage relative to the vehicle control group, which was set as 100%.

The experimental groups were as follows (Applicable to the following experiment):

Control: Fresh medium only; DMSO: Vehicle control, usual medium containing 0.1% DMSO;

Turnagainolide B (B): Medium containing 50 μM turnagainolide B;

VPA: Medium containing 3.3 mM VPA (S161023-25G, Aladdin, Shanghai, China);

CQ: Medium containing 20 μM chloroquine (C193834-1g, Aladdin, China);

B + CQ: Medium containing 50 μM turnagainolide B and 20 μM chloroquine;

B + VPA: Medium containing 50 μM turnagainolide B and 3.3 mM VPA;

B + CQ + VPA: Medium containing 50 μM turnagainolide B, 20 μM chloroquine and 3.3 mM VPA.

### 4.4. Cell Migration

Cell migration ability was evaluated using a standard wound healing (scratch) assay. Briefly, B16-F10 cells were seeded in 6-well plates and cultured until they reached approximately 90–95% confluence to form a uniform monolayer. A sterile 10 μL pipette tip was used to scratch a straight, cell-free line across the center of each well, with about three lines per well of 6 well plate. The detached cells and debris were gently removed by washing with phosphate-buffered saline (PBS) twice. Subsequently, fresh culture medium as mentioned above with the respective treatments was added.

The initial wound (0 h time point) was immediately photographed under an inverted phase-contrast microscope (Mshot, Guangzhou, China). The plates were then returned to the incubator, and the same wound areas were photographed again at 8, 16, 24 h post-scratch. The migratory area was quantified by measuring the cell-free gap width at multiple fixed positions along the scratch using ImageJ 1.51j8 software (National Institutes of Health, USA). The percentage of wound closure at each time point was calculated using the following formula: Wound Closure (%) = [(Gap Width at 0 h − Gap Width at T hours)/Gap Width at 0 h] × 100%. Data presented are from at least three independent experiments.

### 4.5. NO Test

BV2 microglial cells were pretreated with 50 µM turnagainolide B for 1 h, then stimulated with LPS (1 µg/mL) for 24 h. Supernatants were collected and NO content was determined using a colorimetric NitricOxideAssayKit (E1030, Applygen Technologies Inc., Beijing, China). Absorbance was measured at 550 nm, and NO concentration was calculated based on a standard curve. Inhibition of LPS-induced NO release was expressed as a percentage relative to the LPS-only group.

### 4.6. HDAC

HDAC activity was measured using the Amplite™ Fluorimetric HDAC Activity Assay Kit (13601, AAT Bioquest, Pleasanton, CA, USA). MB49 cells were lysed and protein concentration was normalized. Lysates were incubated with test compounds (50–400 µM) or Trichostatin A control (HDAC inhibitor), followed by addition of HDAC Green™ Substrate. Fluorescence was read at Ex/Em = 490/525 nm after 30–60 min incubation at 37 °C. Activity was calculated relative to vehicle control.

### 4.7. Western Blot Analysis

Total cellular proteins were extracted using ice-cold RIPA lysis buffer (20115ES60, Yeasen, China) supplemented with 1% cOmplete™ Protease Inhibitor Cocktail (04693116001, Roche, Basel, Switzerland), and phosphatase inhibitors (20109ES05, Yeasen, China). The lysates were collected, incubated on ice for 30 min with intermittent vortexing, and then centrifuged at 12,000× *g* for 15 min at 4 °C. The supernatant (total protein extract) was collected. Protein concentration was determined using a bicinchoninic acid Pierce™ BCA Protein Assay Kit (23227, Thermo Scientific, Waltham, MA, USA) according to the manufacturer’s protocol. Equal amounts of protein (10–20 μg) were separated by 10–15% SDS-PAGE and transferred onto PVDF membranes (Millipore, Burlington, MA, USA). After blocking with 5% non-fat milk in TBST for 2 h at room temperature, the membranes were incubated overnight at 4 °C with primary antibodies. The following antibodies were used: anti-LC3B (1:1000, T55992, abmart, Shanghai, China), P62/SQSTM1 (1:1500, 12804, SAB, Greenbelt, MD, USA), anti- SQSTM1/P62 (Phospho-Ser403) (1:1500, 12804, SAB), anti-ATG5 (1:2000, 12994T, CST), anti-BNIP3 (1:1000, Selleck Chemicals, Houston, TX, USA), and anti-GAPDH (1:5000, GB11002, ServiceBio, China). After washing, membranes were incubated with HRP-conjugated secondary antibodies (1:5000, RGAR001, proteintech, Rosemont, IL, USA) for 1 h at room temperature. Protein bands were visualized using an enhanced chemiluminescence (ECL) detection kit (Super sensitive ECL luminescence reagent, MA0186-2, MeilunBio, Shanghai, China) and imaged with a chemiluminescence imaging system (Gelview 6000Plus, BLT, Guangzhou, China). After chemiluminescent detection, membranes were stripped of bound antibodies using a low pH stripping buffer (SB-WB007, Share-bio, Shanghai, China) according to the manufacturer’s protocol. Briefly, membranes were incubated in stripping buffer for 15–20 min at room temperature with gentle shaking, then washed three times with TBST (10 min each). Successful removal of antibodies was confirmed by re-exposing the membranes to chemiluminescent substrate. The stripped membranes were then re-blocked and re-probed with another primary antibody as needed. Band intensities were quantified using ImageJ software (National Institutes of Health, USA).

### 4.8. Quantitative Real-Time PCR

Total RNA was extracted from cells using MolPure^®^ Mag16/48 Tissue/Cell TotalRNA Kit (18607ES16, Yeasen, China) and SPARKeasy Cell RNA kit (AC0205-A, Sparkjade, Jinan, China) according to the manufacturer’s protocol. RNA concentration and purity were determined using a NanoDrop spectrophotometer (Thermo NanoDrop ONE, Thermo Fisher, Waltham, MA, USA). cDNA was synthesized from 1 μg of total RNA using the Hifair^®^ AdvanceFast 1st Strand cDNA Synthesis SuperMix for qPCR (11155ES60, Yeasen, China). qPCR was performed on a Qtower Real-Time PCR System (Qtower, Analytik Jena, Jena, Germany) using NovoStart^®^ Universal Fast SYBR qPCR SuperMix (E401-01A, Novoprotein, Shanghai, China). The PCR conditions were as follows: 95 °C for 30 s, followed by 40 cycles of 95 °C for 5 s and 60 °C for 30 s. The primer sequences used were as follows:

mTOR: Forward 5′-TTGGACGGTGTAGAACTTG-3′, Reverse 5′-GAGATGTTGCCTGCTTGA-3′.

BNIP3: Forward 5′-CTTCAGCAATGGCAATGG-3′, Reverse 5′-TGGTATCTTGTGGTGTCTG-3′.

Pik3ca: Forward 5′-ATGCTTGGCTCTGGAATG-3′, Reverse 5′-CTGCTTGATGGTGTGGAA-3′.

Cyp1a1: Forward 5′-CGTGTCAGTAGCCAATGT-3′, Reverse 5′-GTATCCAGAGCCAGTAACC-3′.

Pak3: Forward 5′-TATACTCGCTCCGTGGTT-3′ Reverse 5′-TCTCCTCATCCGTCATCTT-3′.

Aire: Forward 5′-CGTCTGAAGGAGAAGGAAG-3′ Reverse 5′-AAGAGGAAGGTGCTGTGA-3′.

β-actin (internal control): Forward 5′-GCACCACACCTTCTACAA-3′, Reverse 5′-TACGACCAGAGGCATACA-3′.

The relative mRNA expression levels were calculated using the 2^−ΔΔCt^ method and normalized to β-actin.

### 4.9. RNAseq

Three independent batches of B16-F10 cells were prepared with 50 µM turnagainolide B treated for 24 h. Cell pellets were flash frozed by liquid nitrogen for sequencing. Gene expression analysis was performed on the three batches cell line and on multiple selected normal tissues by DESeq2, using gene raw counts as the input with default parameters [58]. Genes with adjusted fold change ≥1.5, *p* < 0.01 were considered to be significant.

### 4.10. Immunofluorescence Staining

B16-F10 cells were seeded onto glass coverslips in 24-well plates with 3 × 10^4^ cells per well, and allowed to adhere overnight. After treatment with medicine as different groups, cells were fixed with 4% paraformaldehyde for 15 min, permeabilized with 0.1% Triton X-100 in PBS for 10 min, and blocked with Instant^TM^ blocking buffer (AFM-B100, Swiss Affinibody LifeScience AG, Shanghai, China) for 20 min at room temperature. Cells were then incubated overnight at 4 °C with primary antibodies against LC3B (1:400, T55992, abmart, China), and P62 (1:400, 56089, SAB, USA). After washing, cells were incubated with Alexa Fluor 488-conjugated secondary antibodies (1:500, 33206ES60, Yeasen, China) and Hoechst 33342 Stain Solution (C0031, Beijing Solarbio Science & Technology Co., Ltd., Beijing, China) for 1 h at room temperature in the dark, with Actin-Tracker Red-Rhodamine (1:200, C2207S, Beyotime Biotech Inc., Shanghai, China) to help catch the cell structure. Hoechst is utilized in primary experiment, while DAPI is for official experiment. Nuclei were counterstained with DAPI within mounting medium of AntiFade Mounting Medium (HY-K1047, MCE, Monmouth Junction, NJ, USA) and store at 4 °C. Images were captured using a confocal laser scanning microscope (A1R, Nikon, Melville, NY, USA) and analyzed with ImageJ to quantify the number and intensity of fluorescent puncta per cell.

### 4.11. Apoptosis Analysis by Flow Cytometry

Apoptosis was assessed using the Annexin V-FITC/PI Apoptosis Detection Kit (40302ES60, Yeasen, China). Briefly, B16-F10 cells were seeded in 6-well plates at a density of 1.5 × 10^5^ cells per well and allowed to adhere overnight. Cells were then treated with 50 μM turnagainolide B or 0.1% DMSO (vehicle control) for 24 h. After treatment, both adherent and floating cells were collected. The cell pellet was washed twice with cold PBS and resuspended in 1× Binding Buffer. According to the manufacturer’s protocol, cells were stained with 5 μL of Annexin V-FITC and 10 μL of Propidium Iodide (PI) for 15 min at room temperature in the dark. Apoptosis was analyzed immediately by a Beckman Flow Cytometer in the FACS facility (CytoFLEX, Beckman, Brea, CA, USA). Data from at least three independent experiments were analyzed using PRISM GraphPad 9 (version 9.0.0) software. 

### 4.12. In Vivo Tumor Formation Inhibition Assay

Female C57BL/6J mice (6–8 weeks old) were used for the syngeneic tumor model. B16-F10 cells (2 × 10^5^ cells) were suspended with 100 μL B solution (2 mM) and subcutaneously injected into the right flank of each mouse. Mice were randomly divided into two groups (*n* = 6 per group): (1) Vehicle control group (0.1% DMSO in saline), (2) turnagainolide B treatment group (50 μM). Tumor dimensions (length and width) and mouse body weights were measured every three days. Tumor volume was calculated using the formula: Volume = 0.5 × length × width^2^. On the 19th day of the experiment, mice were euthanized, tumors were excised and photographed. Portions of tumor tissues were fixed in 4% paraformaldehyde for histological examination (H&E staining, immunohistochemistry). All mice were handled by the Association for Assessment and Accreditation of Laboratory Animals Care international guidelines. Animal protocols were approved by the Institutional Animal Care and Use Committee (IACUC), Shenzhen Institute of Advanced Technology (SIAT), Chinese Academy of Science (SIAT-IACUC-230718-HCS-HJD-A2300).

### 4.13. Nanolive Live-Cell Imaging for Real-Time Morphology and Death Analysis

Real-time, label-free imaging of cellular morphological dynamics was conducted using a Nanolive 3D Cell Explorer fluorescence microscope (Nanolive, Lausanne, Switzerland). B16-F10 cells were seeded in ibdi μ-Dishes for confocal microscopy (35 mm, glass bottom; ibidi GmbH, Gräfelfing, Germany) and allowed to adhere overnight. Prior to imaging, the medium was replaced with fresh medium containing either 50 μM turnagainolide B or 0.1% DMSO (vehicle control). The dish was then transferred to the microscope stage equipped with a stage-top incubation system maintaining a constant environment of 37 °C, 5% CO_2_, and controlled humidity. For each experiment, a field of view containing 3–10 cells was selected and subjected to continuous time-lapse holotomographic imaging for 24 h, with a three-dimensional refractive index map acquired at from 30 s to 15 min intervals respectively. Image processing and analysis of morphological changes were performed using Nanolive STEVE FULL (version 1.6.3496) software and imageJ.

### 4.14. AI Prediction and Molecular Docking

To gain preliminary insight into the potential molecular targets of turnagainolide B, an in silico prediction was performed using the SwissTargetPrediction web tool (http://www.swisstargetprediction.ch/). The canonical SMILES notation of turnagainolide B was used as the query input:

(CC[C@H](C)[C@H]1C(=O)N[C@H](C(=O)O[C@@H](CC(=O)N[C@H](C(=O)N[C@@H](C(=O)N1)C)C(C)C)/C=C/C2=CC=CC=C2)C(C)C)

The prediction was run under the “Mus musculus” organism setting, and the results were ranked based on the calculated probability score (Supporting Table).

The three-dimensional structure of the PI3Kγ was retrieved from the Protein Data Bank (ID: 3L54; resolution, 2.30 Å; http://www.rcsb.org/ (accessed on 22 April 2026)). For docking analysis, all protein and molecular files were converted into PDBQT format with all water molecules excluded and polar hydrogen atoms were added. The grid box was centered to cover the domain of each protein and to accommodate free molecular movement. The grid point distance was 0.05 nm, and grid box was set to 30 Å × 30 Å × 30 [30]. Molecular docking was carried out using AutoDock (Version 4.2) and MOE_2019.0102 (https://www.chemcomp.com/ accessed on 20 April 2026).

### 4.15. Data Analysis

All experiments were performed at least in triplicate, analyzed by PRISM GraphPad software. Data are presented as mean ± Standard Error of Mean (SEM). Statistical significance was determined using one-way or two-way ANOVA followed by Tukey’s post hoc test. A *p*-value of less than 0.05 was considered statistically significant. (* *p*  <  0.05, ** *p*  <  0.01, *** *p*  <  0.001, **** *p*  <  0.0001).

## 5. Conclusions

In this study, bioassay-guided isolation from the marine soil sediment-derived bacterium *B. subtilis* LP led to the identification of the known cyclic peptide turnagainolide B and a new analogue, turnagainolide H. Their structures were elucidated by comprehensive HRESIMS and NMR analyses, with turnagainolide H identified as a Gly-3-substituted variant of turnagainolide B. Biosynthetic investigation revealed that the *tur* gene cluster generates structural diversity through adenylation-domain substrate promiscuity, whereas a stereoselective KR domain maintains the conserved *S*-configuration at the Hppa C-3 position, a structural feature critical for SHIP1 binding. The discovery of turnagainolide H expands the structural diversity of the turnagainolide family and provides a genetic basis for future biosynthetic engineering.

Functionally, turnagainolide B exhibited potent anti-melanoma activity by inducing a unique dysregulation of autophagic flux characterized by simultaneous autophagy initiation and degradation blockade. This mechanism, which differs from that of classical autophagy inhibitors, effectively suppressed melanoma cell proliferation and migration, with anti-tumor and immunomodulatory effects validated in both in vitro and in vivo models.

Collectively, this work expands the chemical diversity of the turnagainolide family, provides biosynthetic insights, and establishes turnagainolide B as a promising lead scaffold for developing new autophagy-based melanoma therapies that bypass conventional apoptosis pathways and may overcome resistance to existing treatments.

## Figures and Tables

**Table 1:** The ^1^H and ^13^C NMR data of turnagainolides B (**1**) and H (**2**) in DMSO-*d_6_*.

Residue	Position	1 δ_C_	1 δ_H_	2 δ_C_	2 δ_H_
Hppa-1	1	168.75		169.06	
	2	40.06	2.88, dd (14.4, 11.4) 2.42, dd (14.4, 2.4)	40.06	2.90, dd (14.8, 11.4) 2.45, dd (14.8, 2.2)
	3	73.03	5.50, dd (13.6, 7.2)	72.76	5.55, dd (11.3, 7.4)
	4	126.73	6.28, dd (16.0, 7.2)	126.78	6.27, dd (16.0, 7.0)
	5	132.51	6.68, d (16.0)	132.54	6.67, d (16.0)
	6	135.76		135.79	
	7,11	126.56	7.44, d (7.6)	126.60	7.44, d (7.6)
	8,10	128.74	7.35, t (7.6)	128.82	7.35, t (7.6)
	9	128.19	7.28, t (7.6)	128.26	7.28, t (7.6)
Val-2	1	172.36		172.66	
	2	57.53	4.14, dd (8.6, 6.8)	58.13	4.06, m
	3	29.88	1.96, m	29.66	1.98, dd (13.6, 6.8)
	4	19.41	0.89, dd (9.4, 4.8)	19.49	0.88, m
	4′	19.65	0.89, dd (9.4, 4.8)	19.47	0.88, m
	NH		7.74, d (8.5)		7.87, d (8.2)
Ala-3 (Gly-3)	1	173.07		169.80	
	2	48.84	4.32, p (6.8)	43.56	3.38, m 3.99, dd (14.2,4.8)
	3	16.37	1.18, d (7.0)		
	NH		8.56, d (5.8)		8.71, t (5.8)
Ile-4	1	170.43		170.46	
	2	57.22	4.26, m	58.41	4.24, d (9.4)
	3	35.57	2.05, m	35.80	2.01, m
	4	11.95	0.81, m	11.84	0.82, m
	4′	23.55	1.25, m	20.85	1.22, m
	5	15.51	0.81, m	15.69	0.82, m
	NH		8.10, d (9.6)		8.20, d (9.8)
Val-5	1	168.72		168.74	
	2	58.28	4.24, m	57.91	4.20, dd (9.4,5.2)
	3	28.48	2.25, m	28.99	2.20, m,
	4	18.93	0.89, m	18.97	0.88, m
	4′	18.27	0.89, m	18.27	0.88, m
	NH		7.59, d (9.6)		7.67, d (9.4)

## Data Availability

The original contributions presented in this study are included in the article.

## Supplementary Materials

The following supporting information can be downloaded at: https://www.mdpi.com/article/10.3390/md24070235/s1, Supplementary_figures and table (PDF), Supplementary_video (mp4). Figure S1: The UV of Turnagainolide B and H; Figure S2: The HRESIMS spectrum of Turnagainolide B and H; Figure S3: NMR spectrum of Turnagainolide B (**1**); Figure S4: NMR spectrum of Turnagainolide H (**2**); Figure S5: Determination of the chiral configuration of amino acid residues; Figure S6: ^1^H NMR spectrum of (*S*)-MPTA ester derivatization of Turnagainolide H (**2**) recorded at 600 MHz in chloroform-*d*; Figure S7: ^1^H NMR spectrum of (*R*)-MPTA ester derivatization of Turnagainolide H (**2**) recorded at 600 MHz in chloroform-*d*; Figure S8: Extraction and MTT test of three Turnagainolide isolated isomers; Figure S9: Functional assay of apoptosis; Figure S10: Phase contrast microscope photos of B16-F10 after 24 h; Figure S11: RNAseq: KEGG and GO; Figure S12: qPCR results related to cell migration, tumor immunology and autophagy (2~4 h); Figure S13: Animal experiment: mouse photo and weight; Figure S14: The chemical structures of turnagainolides family; Table S1: Supporting Table. Predicted targets of Turnagainolide B using SwissTargetPrediction, highlighting PIK3CG-p110; Video S1: Supplementary_video. Real-time Nanolive holotomographic imaging of B16-F10 melanoma cells treated with turnagainolide B.

## Abbreviations

The following abbreviations are used in this manuscript:

AKT	Protein Kinase B
ANOVA	Analysis of variance
ATCC	American Type Culture Collection
ATG5	Autophagy related 5
BNIP3	BCL2 interacting protein 3
BSA	Bovine serum albumin
BV2	Mouse microglial cell line
CD4	Cluster of differentiation 4
CD8	Cluster of differentiation 8
cDNA	Complementary DNA
CQ	Chloroquine
COSY	Correlation spectroscopy
CST	Cell Signaling Technology
DAPI	4′,6-diamidino-2-phenylindole
DEG	Differential Expression Gene
DMEM	Dulbecco’s modified Eagle medium
DMSO	Dimethyl sulfoxide
DNA	Deoxyribonucleic acid
ECL	Enhanced chemiluminescence
EDTA	Ethylenediaminetetraacetic acid
EtOAc	Ethyl acetate
FACS	Fluorescence activated cell sorting
FBS	Fetal bovine serum
GAPDH	Glyceraldehyde-3-phosphate dehydrogenase
H&E stain	Hematoxylin and eosin stain
HaCaT	Human keratinocyte cell line
HDAC	Histone deacetylase
HPLC	High performance liquid chromatography
Hppa	3-hydroxy-4-pentenoic acid
HRESIMS	High-resolution electrospray ionization mass spectrometry
HRP	Horseradish peroxidase
HSQC	Heteronuclear single quantum coherence
I_C_50	Half maximal inhibitory concentration
IACUC	Institutional Animal Care and Use Committee
IF	immunofluorescence
IHC	Immunohistochemistry
Ile	Isoleucine
LC3B	Microtubule-associated protein 1 light chain 3 beta
LLC	Lewis lung carcinoma
LPS	Lipopolysaccharide
mRNA	Messenger RNA
MB49	Mouse bladder cancer cell line
MEF	Mouse embryonic fibroblast
MeCN	Acetonitrile
MeOH	Methanol
MSC	Mesenchymal stem cell
mTOR	Mammalian target of rapamycin
MTPA	α-methoxy-α-(trifluoromethyl)phenylacetic acid
MTT	3-(4,5-dimethylthiazol-2-yl)-2,5-diphenyltetrazolium bromide
NMR	Nuclear magnetic resonance
NO	Nitric oxide
Panc02	Mouse pancreatic cancer cell line
PBS	Phosphate-buffered saline
PCR	Polymerase chain reaction
PI	Propidium iodide
PI3K	Phosphoinositide 3-kinase
PVDF	Polyvinylidene fluoride
qPCR	Quantitative Polymerase Chain Reaction
RNA-seq	RNA sequencing
SDS-PAGE	Sodium dodecyl sulfate-polyacrylamide gel electrophoresis
SEM	Standard error of the mean
SHIP1	Src homology 2 domain-containing inositol phosphatase 1
Val	Valine
VPA	Valproic acid
