# Supplementary material for: The Marine-Derived Cyclopentapeptide Turnagainolide B Suppresses Melanoma via Autophagic Flux Disruption and Inhibits Tumorigenesis In Vivo
Source: Mar Drugs. 2026 Jul 3;24(7):235. doi: 10.3390/md24070235 (PMC13412985; doi:10.3390/md24070235)
Supplement: Supplementary file 1 [file marinedrugs-24-00235-s001.zip › Supplementary_figures and table.pdf]

Supporting Information for

Original article

The Marine-Derived Cyclopentapeptide Turnagainolide B Suppresses Melanoma via Autophagic Flux Disruption and Inhibits Tumorigenesis In Vivo

Guoyue Wan[a,b,⊥], keyu Zhao[c,⊥], Min Wang[d], Ren-HeXu[a], Meiling Jin[b*], Liwei Liu[e*]

[a]*Center of Reproduction, Development & Aging, Cancer Center and Institute of Translational Medicine, Faculty of Health Sciences, University of Macau, Taipa, Macau 999078, China*

[b]*State Key Laboratory of Quantitative Synthetic Biology, Shenzhen Institute of Synthetic Biology, Shenzhen Institutes of Advanced Technology, Chinese Academy of Sciences, Shenzhen 518000, China*

[c]*Department of Dermatology, The First Affiliated Hospital of Ningbo University, Ningbo 315010, China*

[d]*College of New Materials and Chemical Engineering, Beijing Institute of Petrochemical Technology, Beijing 100000, China*

[e] *Li Dak Sum Yip Yio Chin Kenneth Li Marine Biopharmaceutical Research Center, Health Science Center, Ningbo University, Ningbo 315000, China*

[⊥]Guoyue Wan and Keyu Zhao contributed equally

*Corresponding author (s).

E-mail addresses: ml.jin@siat.ac.cn (Meiling Jin) & liuliwei@nbu.edu.cn (Liwei Liu)

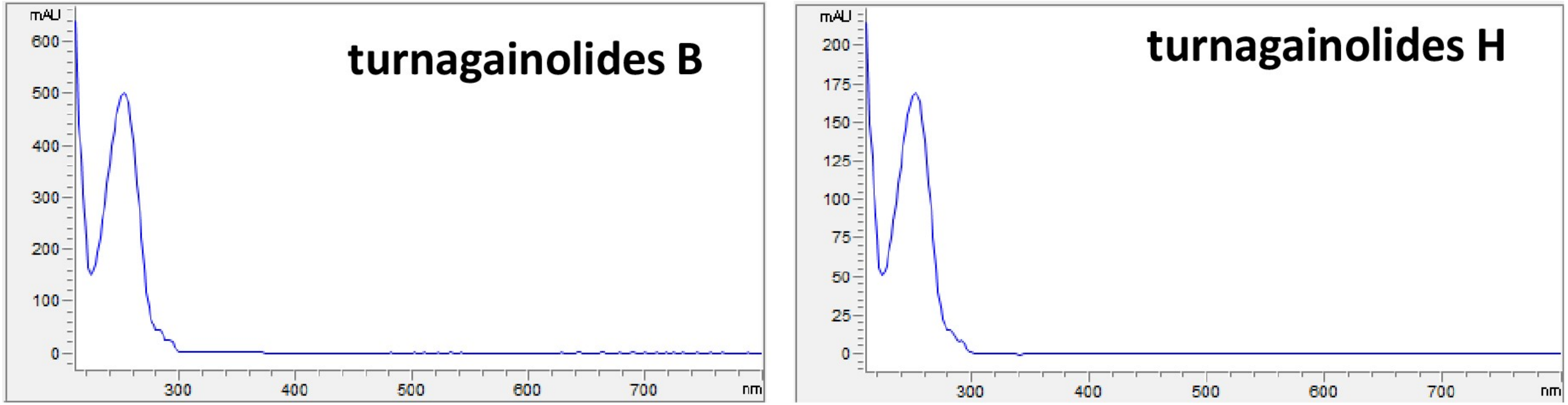

Figure S1. The UV of Turnagainolide B and H

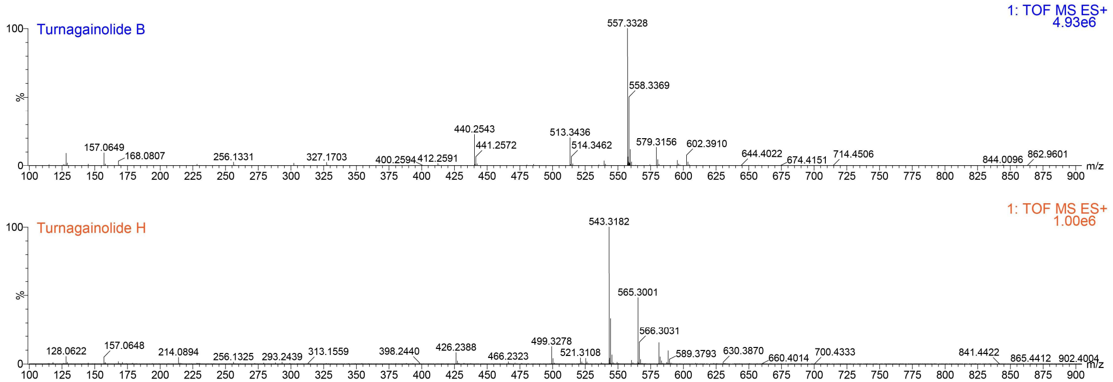

Figure S2. The HRESIMS spectrum of Turnagainolide B and H

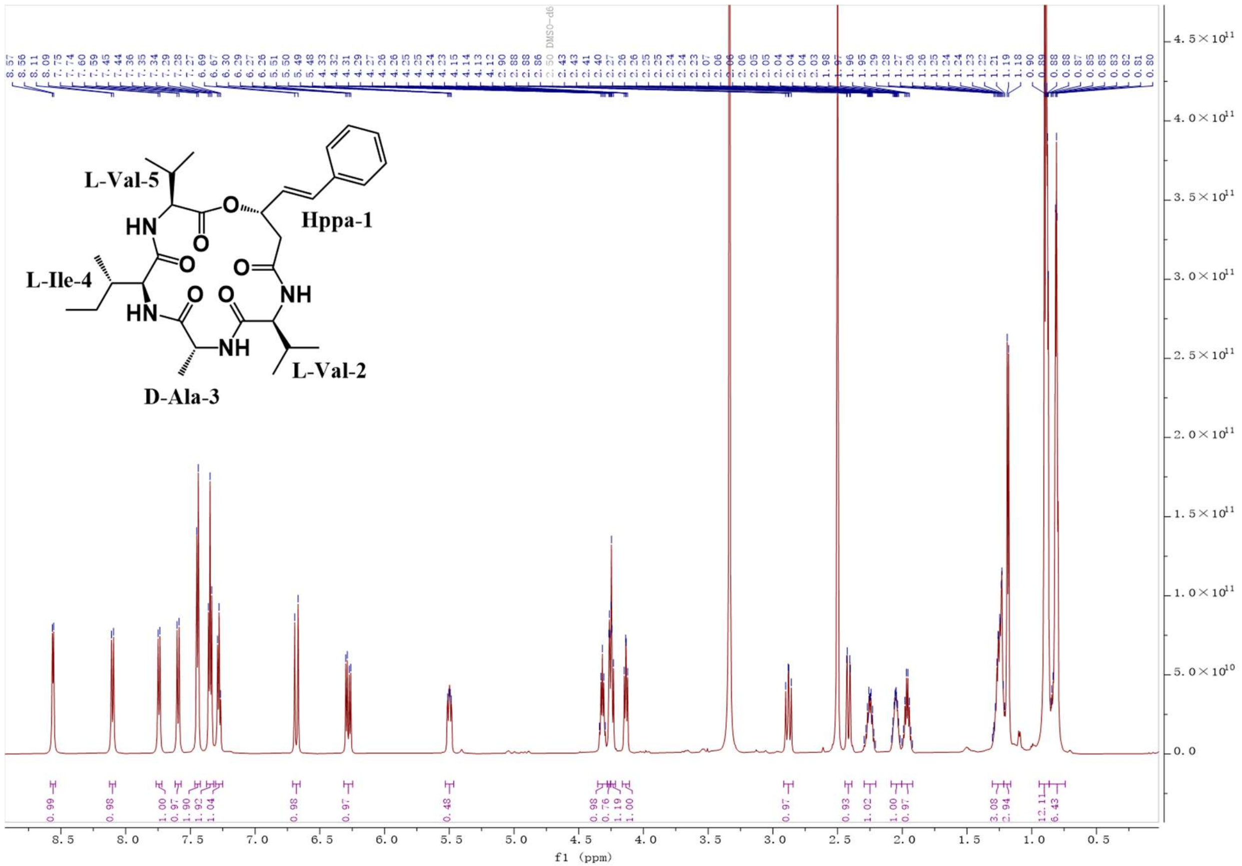

A) ^{1}H NMR spectrum of Turnagainolide B **(1)** recorded at 600 MHz in DMSO-d_6

Figure S3. NMR spectrum of Turnagainolide B **(1)**

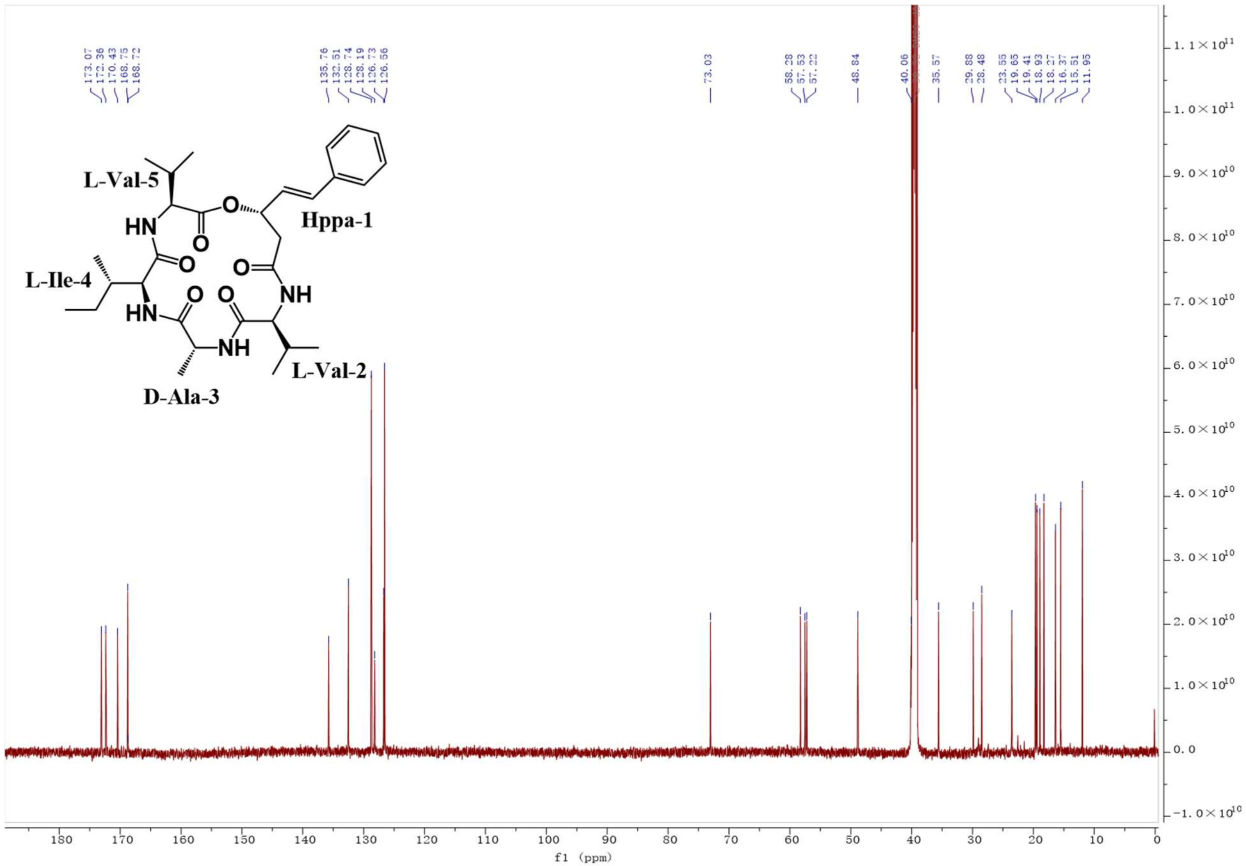

B) 13C NMR spectrum of Turnagainolide B **(1)** recorded at 600 MHz in DMSO-d6

Figure S3. NMR spectrum of Turnagainolide B **(1)**

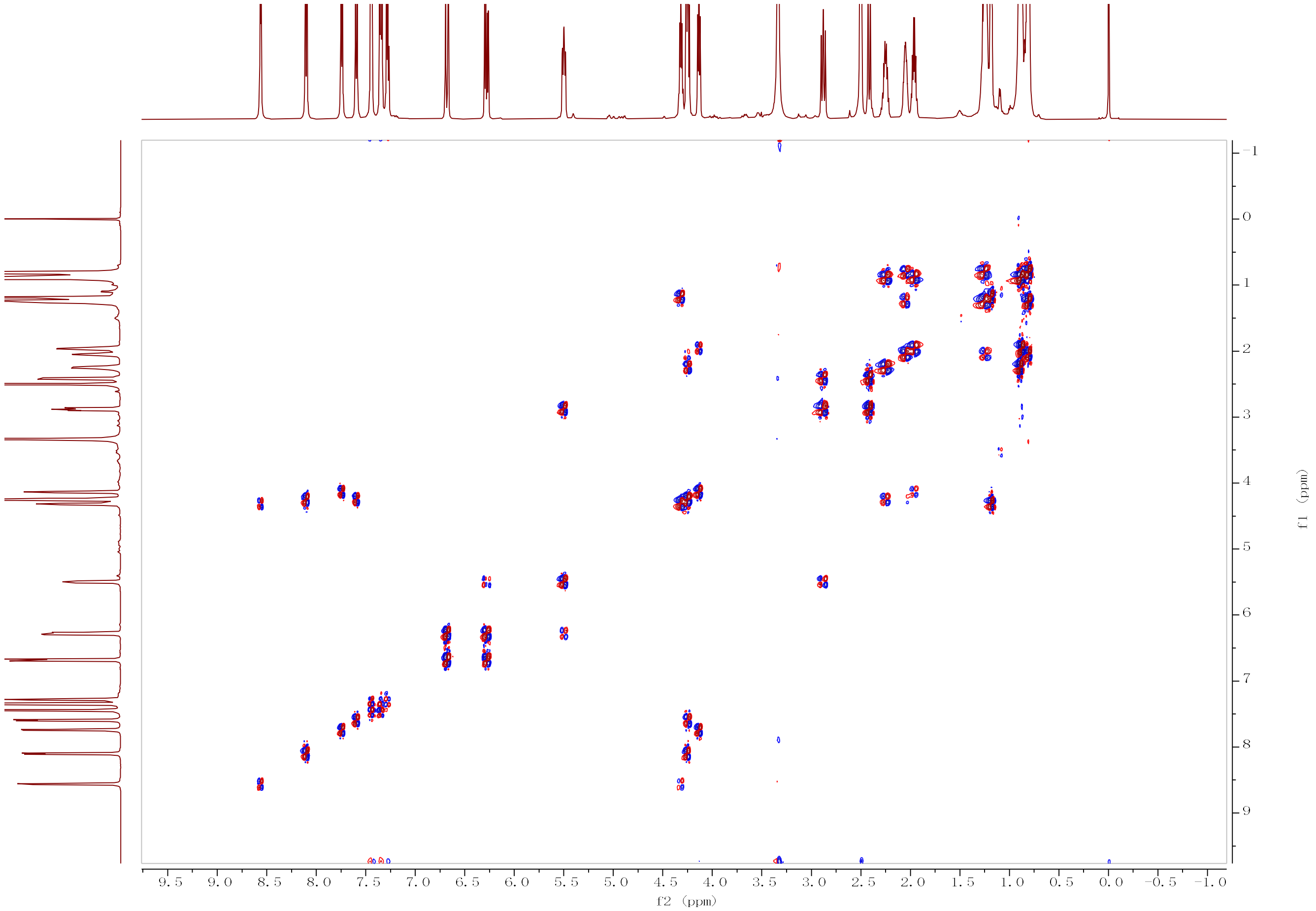

C) COSY NMR spectrum of Turnagainolide B **(1)** recorded at 600 MHz in DMSO-d6

Figure S3. NMR spectrum of Turnagainolide B **(1)**

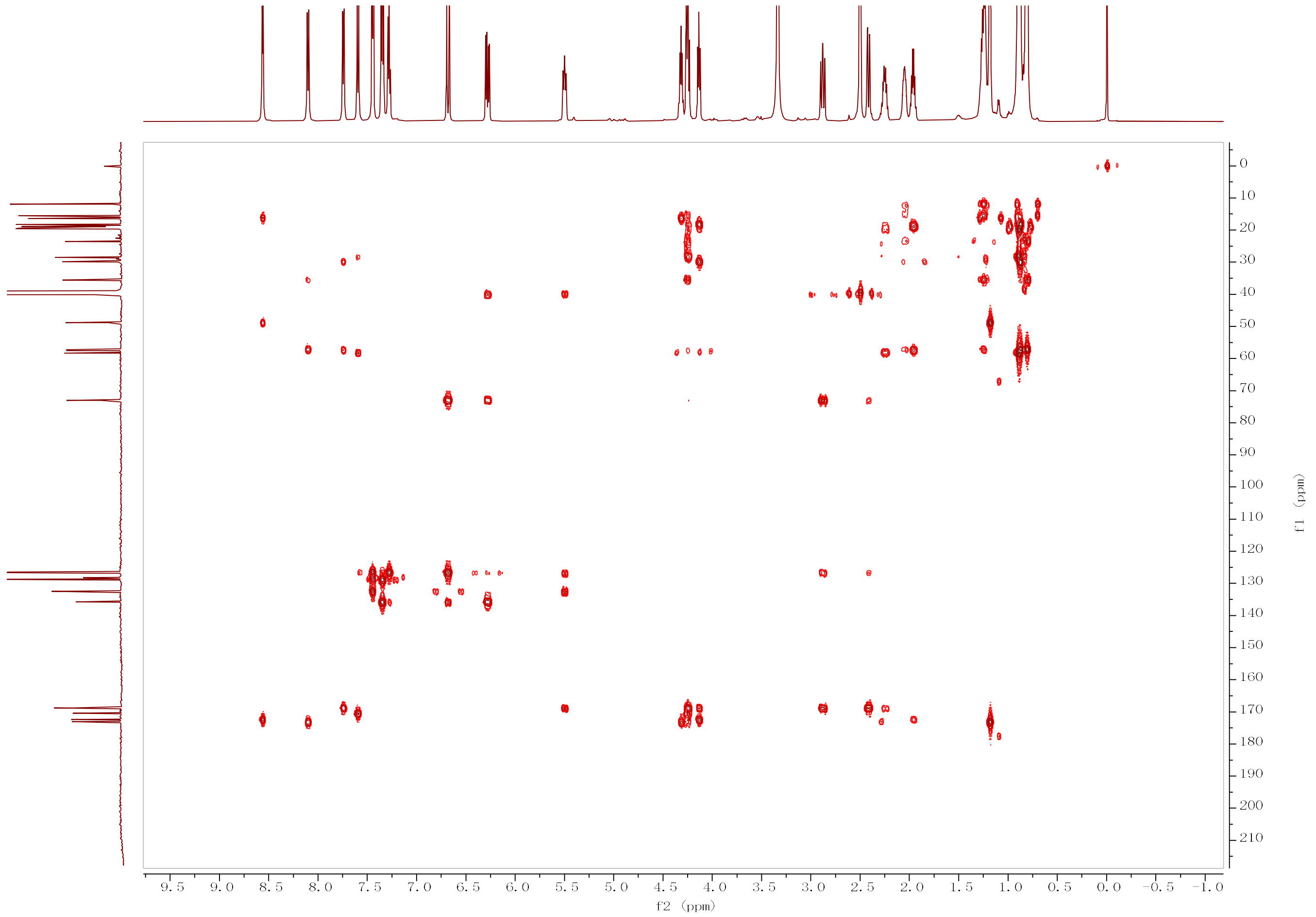

D) HMBC NMR spectrum of Turnagainolide B **(1)** recorded at 600 MHz in DMSO-d6

Figure S3. NMR spectrum of Turnagainolide B **(1)**

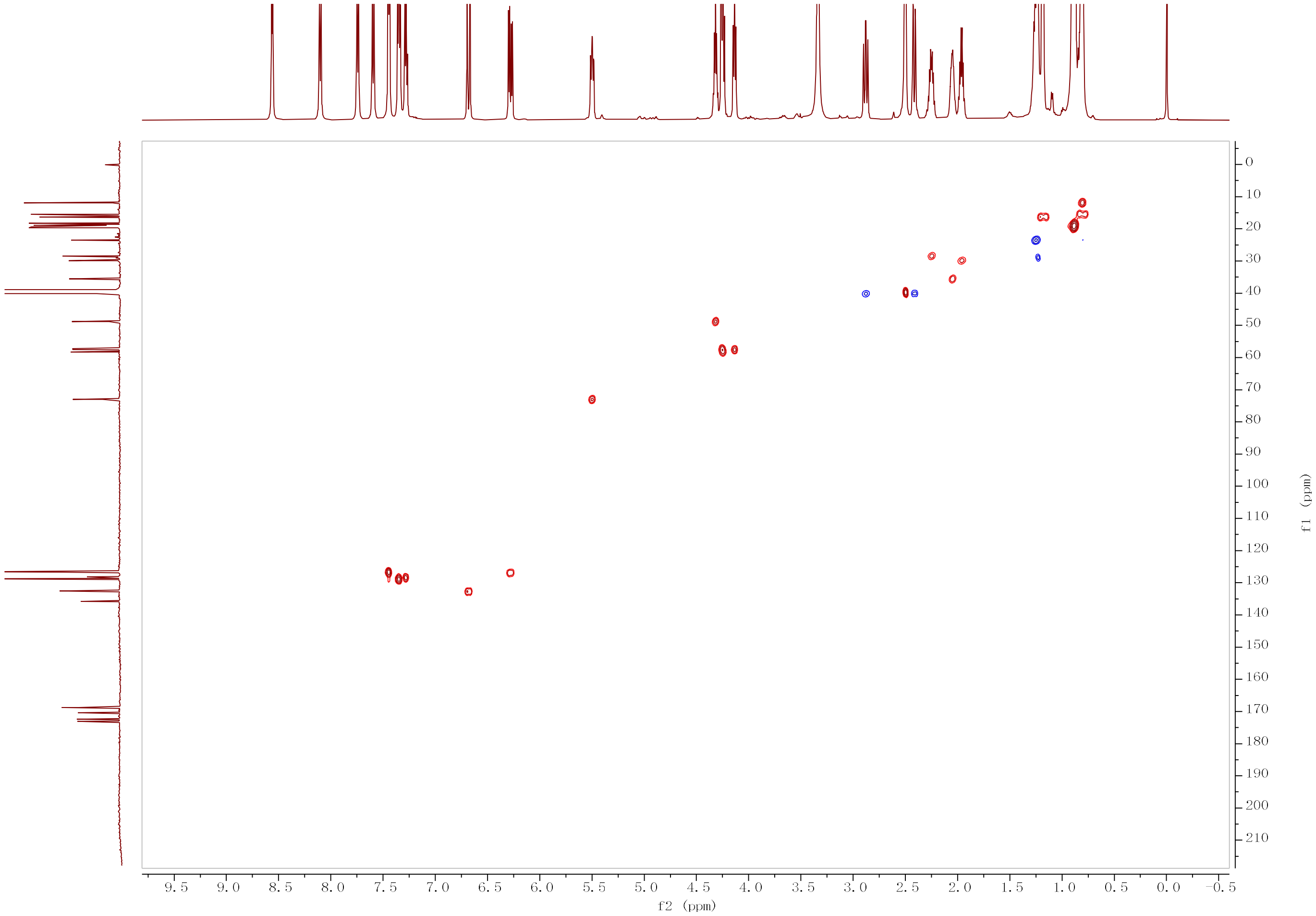

E) HSQC NMR spectrum of Turnagainolide B **(1)** recorded at 600 MHz in DMSO-d_6

Figure S3. NMR spectrum of Turnagainolide B **(1)**

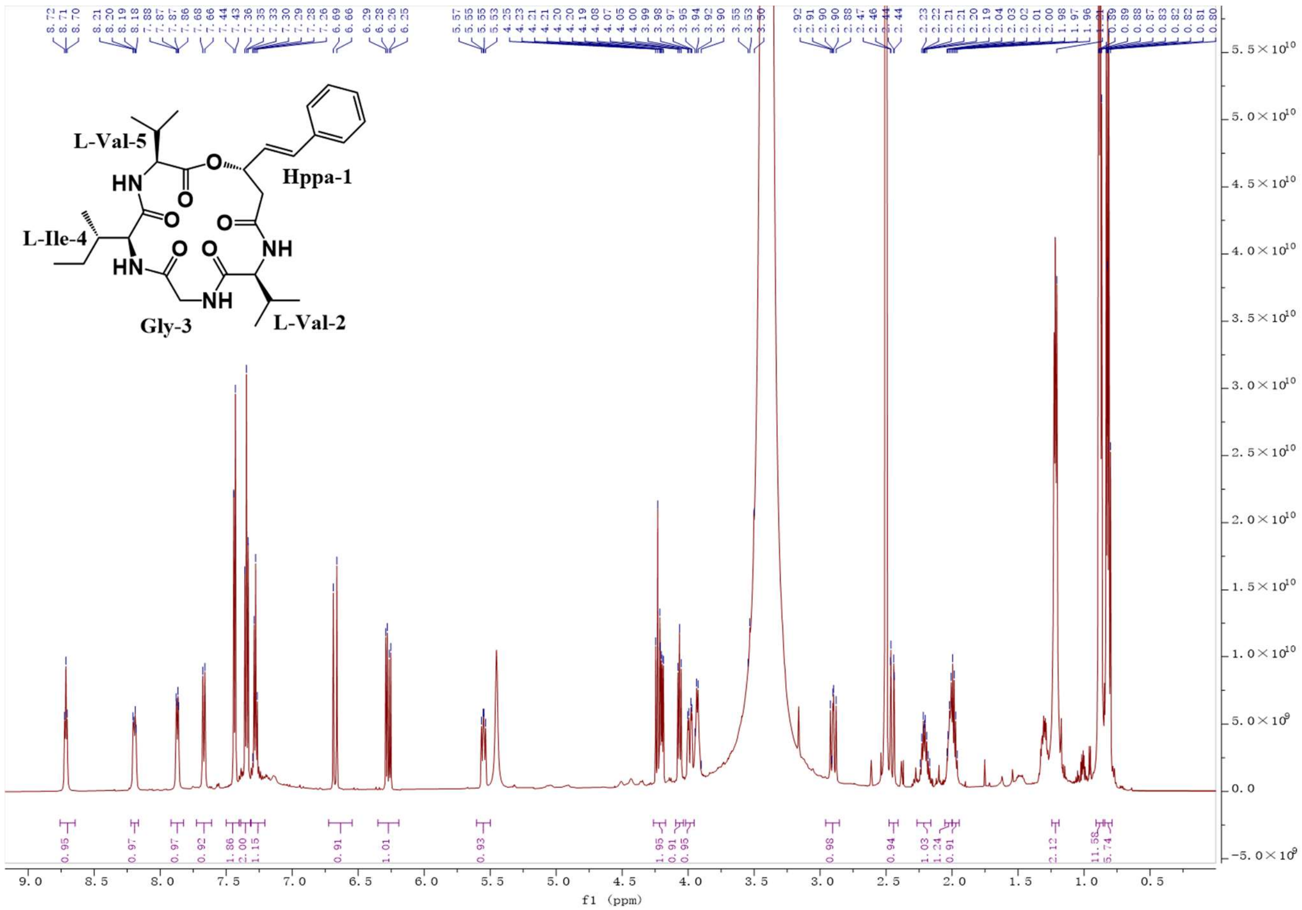

A) ^{1}H NMR spectrum of Turnagainolide H **(2)** recorded at 600 MHz in DMSO-d_6

Figure S4. NMR spectrum of Turnagainolide H **(2)**

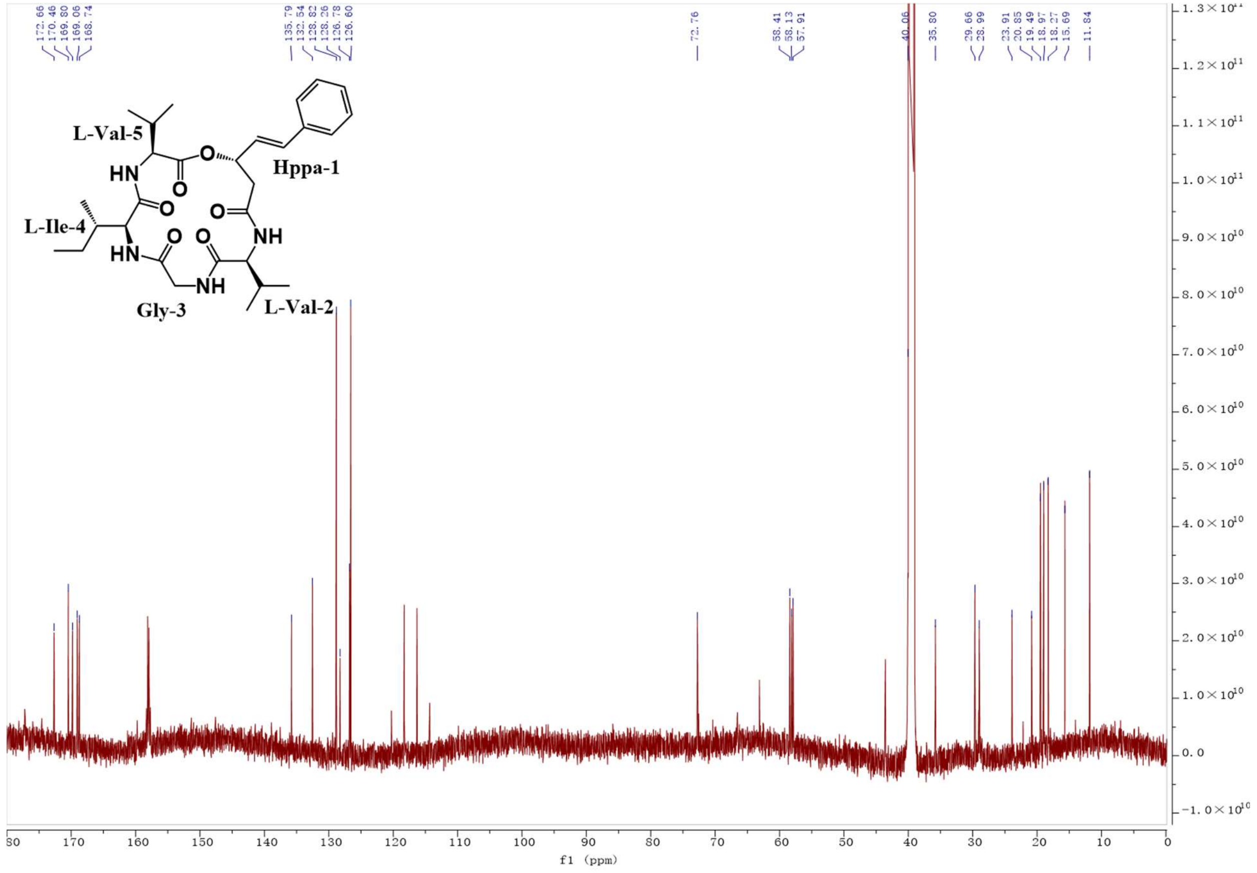

B) ^{13}C NMR spectrum of Turnagainolide H **(2)** recorded at 600 MHz in DMSO-d_6

Figure S4. NMR spectrum of Turnagainolide H **(2)**

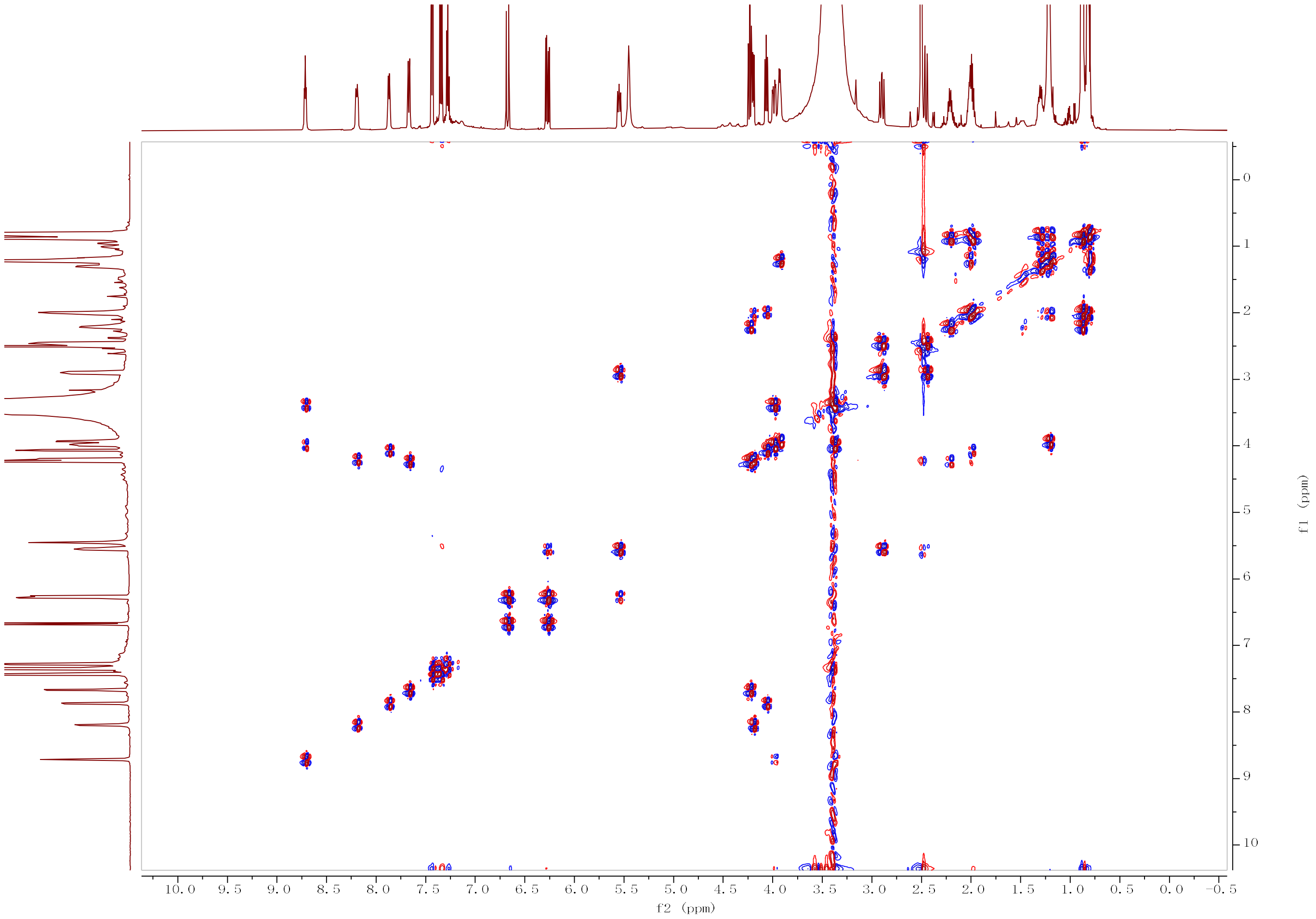

C) COSY NMR spectrum of Turnagainolide H **(2)** recorded at 600 MHz in DMSO-d_6

Figure S4. NMR spectrum of Turnagainolide H **(2)**

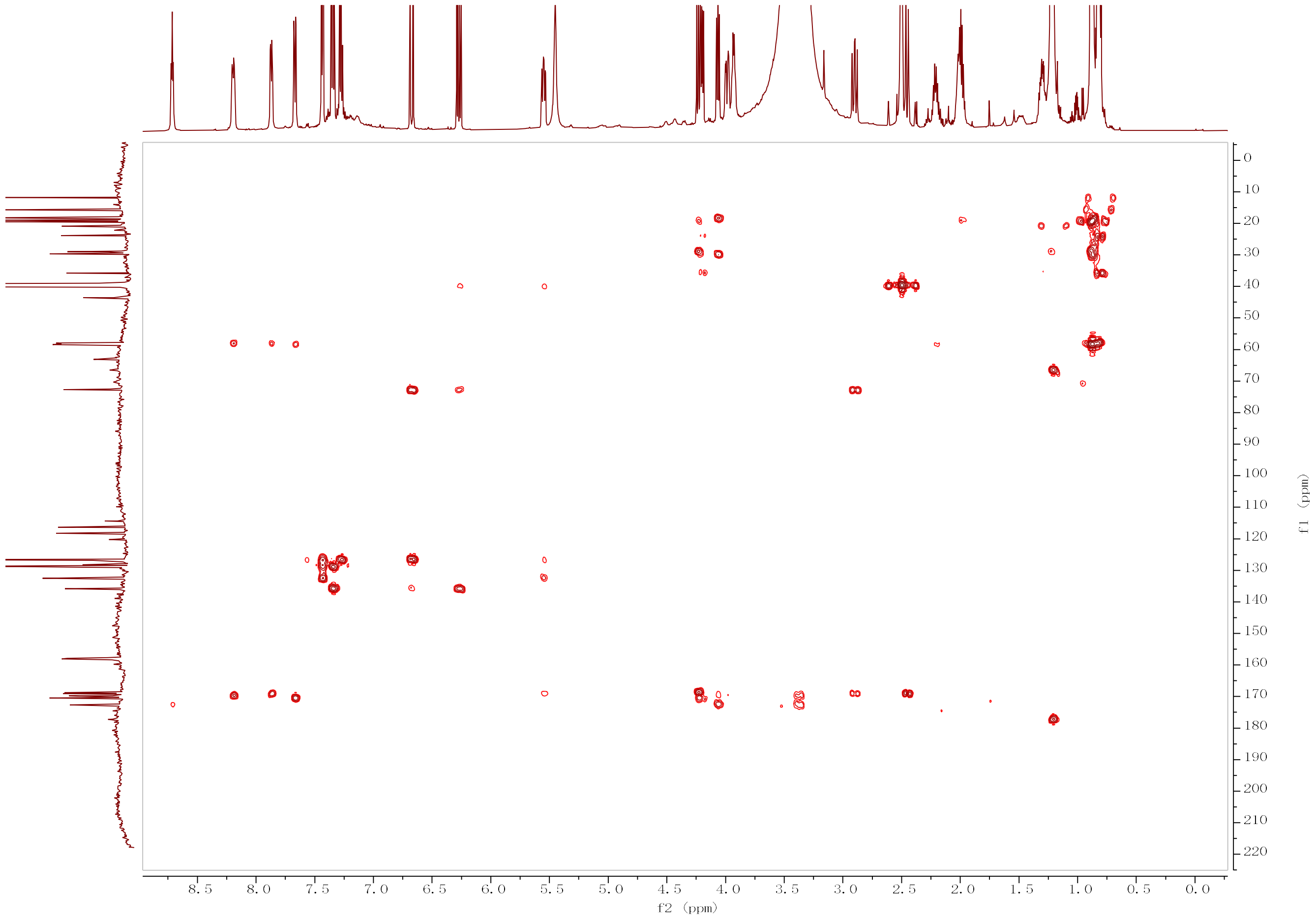

D) HMBC NMR spectrum of Turnagainolide H **(2)** recorded at 600 MHz in DMSO-d_6

Figure S4. NMR spectrum of Turnagainolide H **(2)**

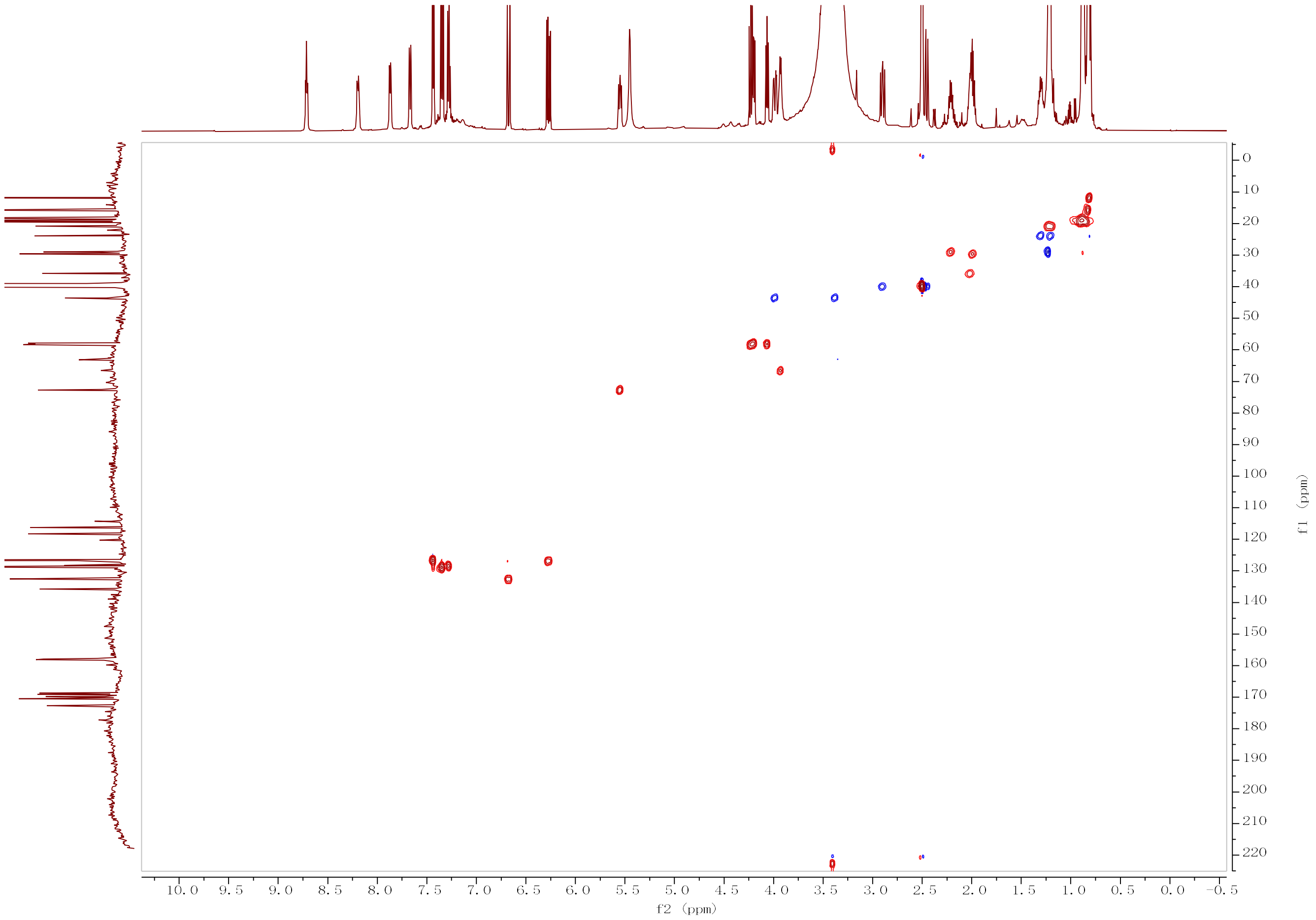

E) HSQC NMR spectrum of Turnagainolide H **(2)** recorded at 600 MHz in DMSO-d_6

Figure S4. NMR spectrum of Turnagainolide H **(2)**

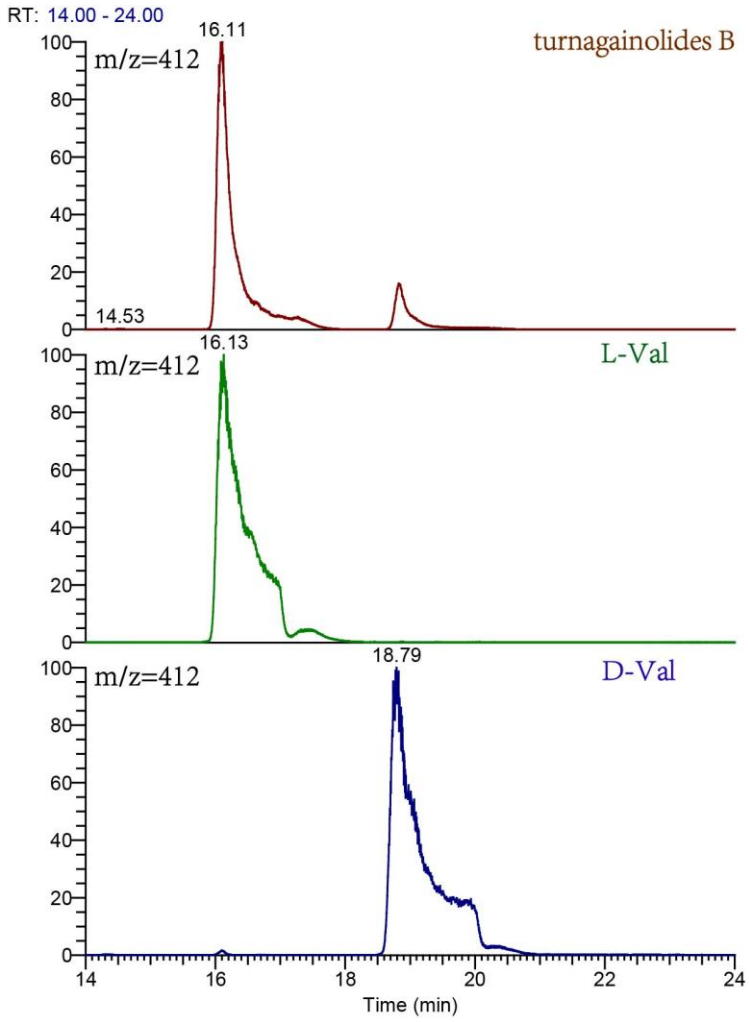

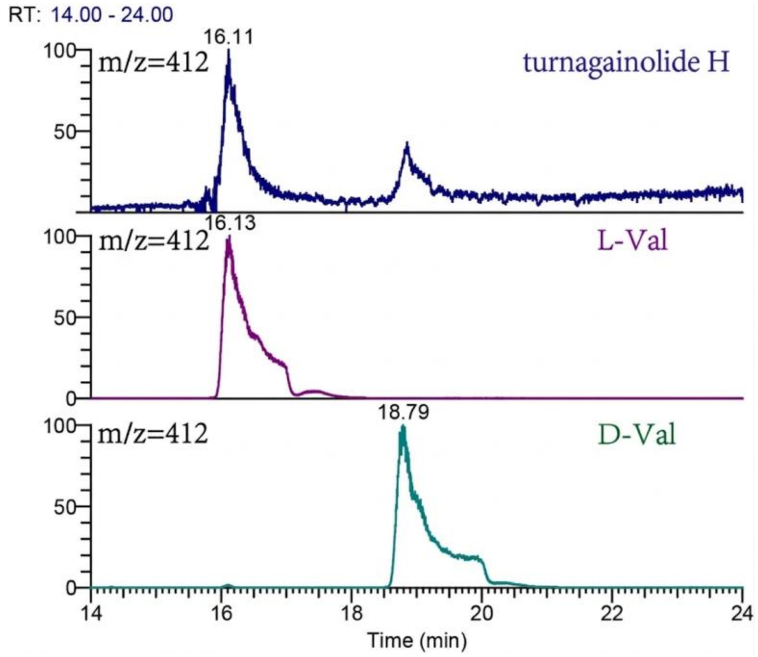

A) Determination of the configuration of Val residues

Figure S5. Determination of the chiral configuration of amino acid residues

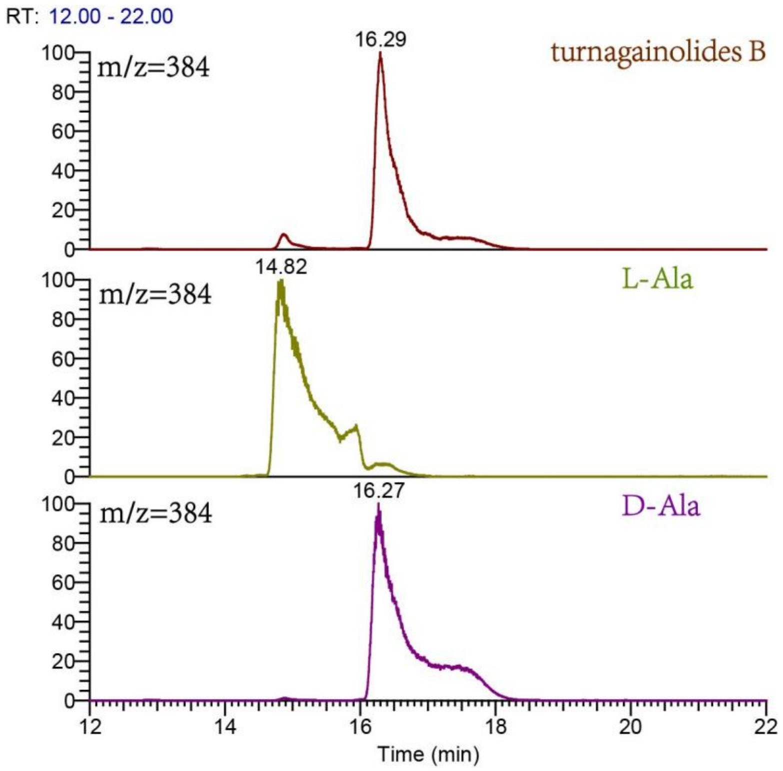

B) Determination of the configuration of Ala residues

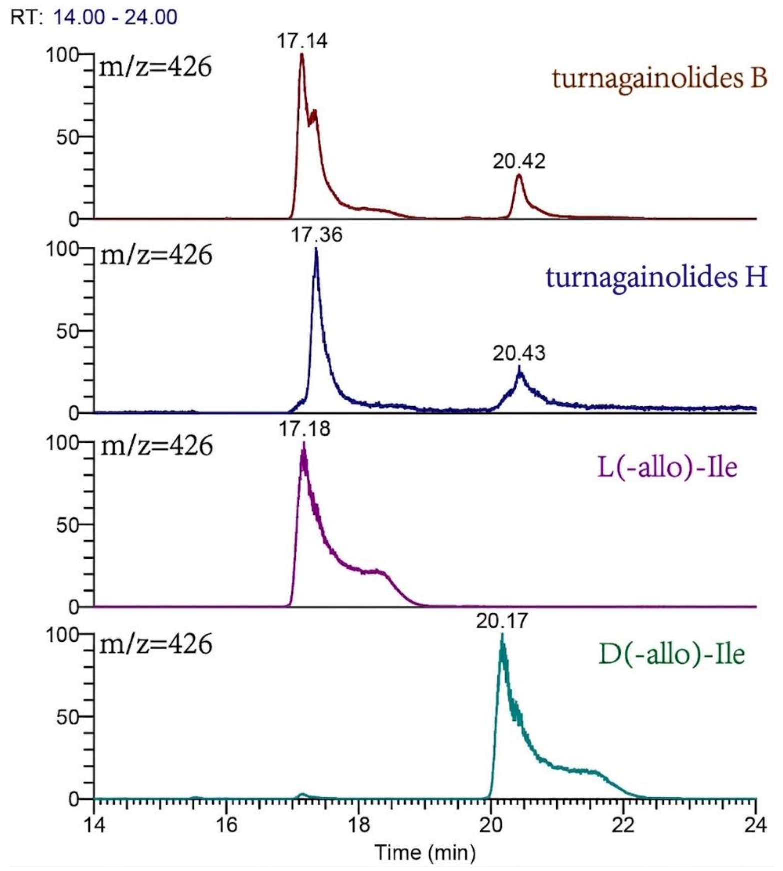

C) Preliminary analysis of Ile residues

Figure S5. Determination of the chiral configuration of amino acid residues

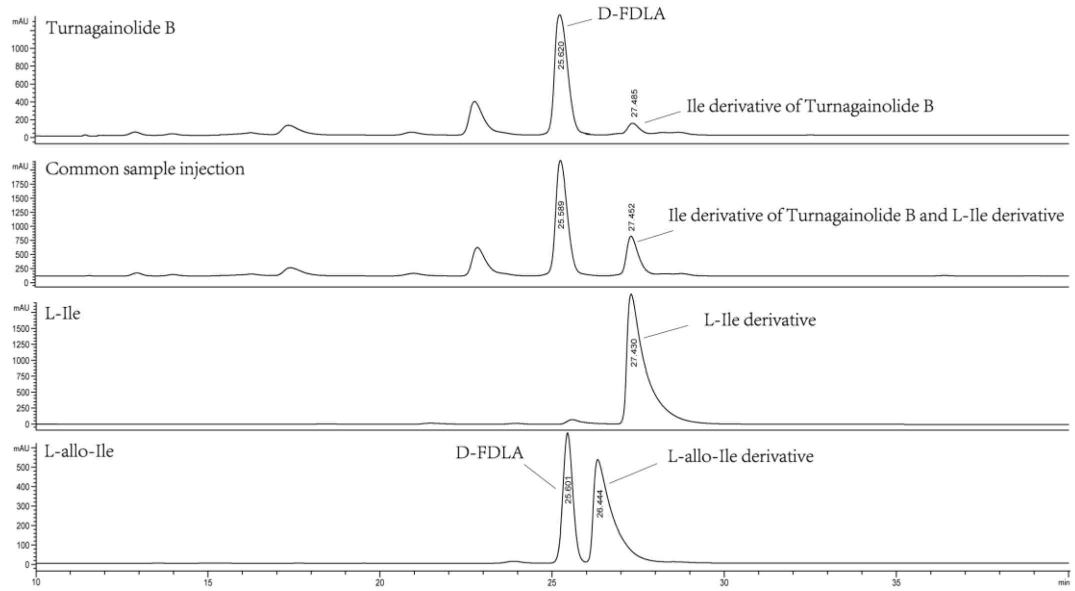

D) Determination of the configuration of Ile residues in Turnagainolide B

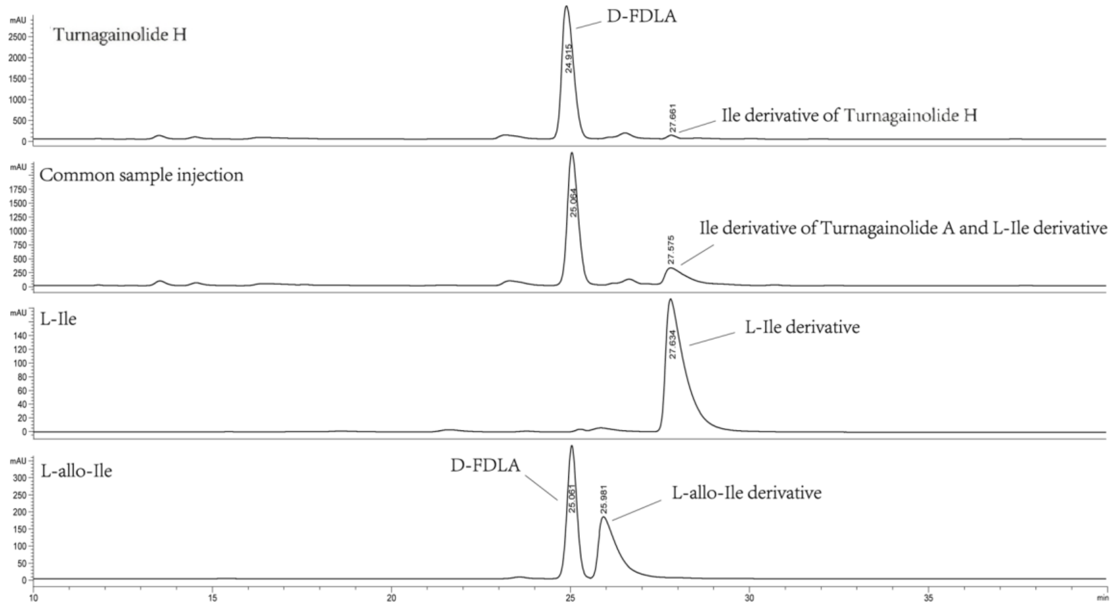

E) Determination of the configuration of Ile residues in Turnagainolide H

Figure S5. Determination of the chiral configuration of amino acid residues

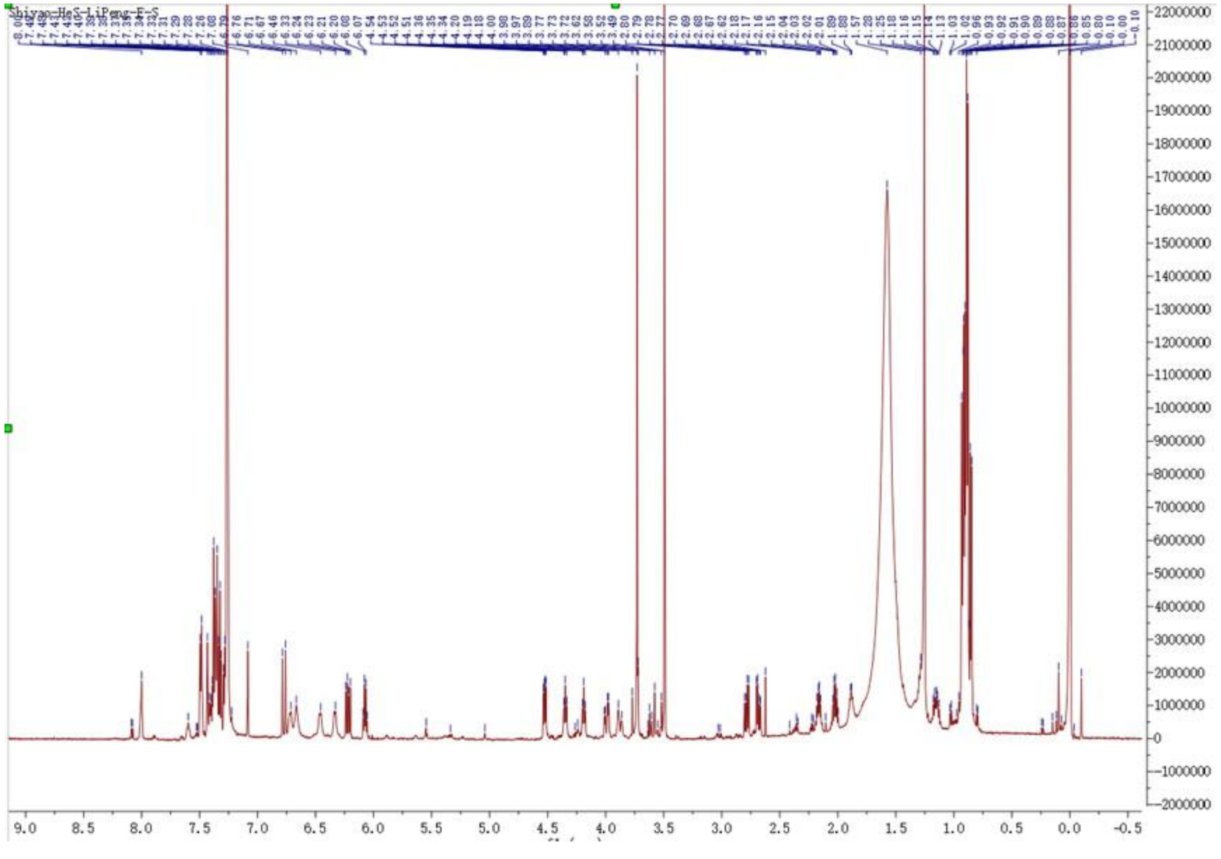

Figure S6. ^{1}H NMR spectrum of (*S*) -MPTA ester derivatization of Turnagainolide H (**2**) recorded at 600 MHz in chloroform-*d.*

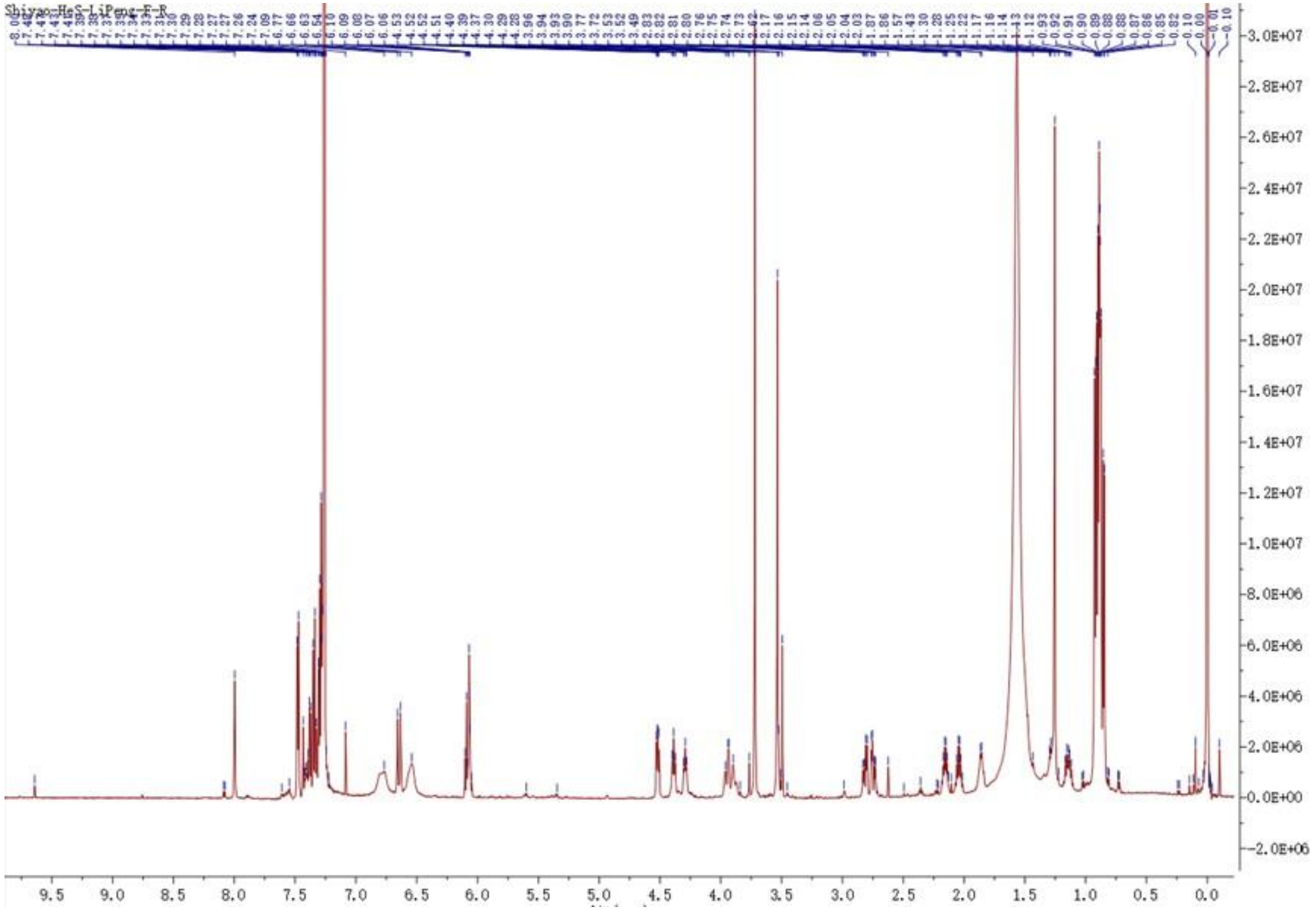

Figure S7. ^{1}H NMR spectrum of (*R*) -MPTA ester derivatization of Turnagainolide H (**2**) recorded at 600 MHz in chloroform-*d.*

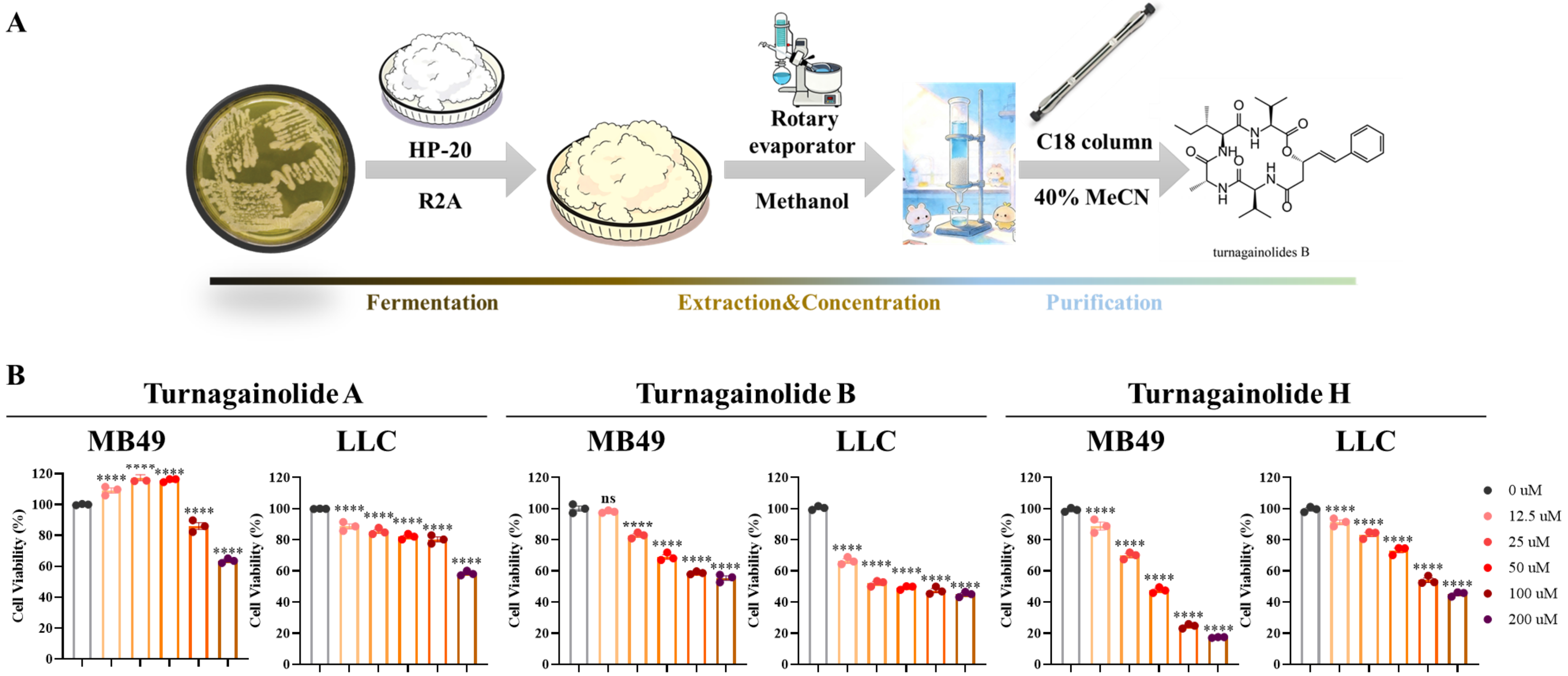

Figure S8. Extraction and MTT test of three Turnagainolide isolated isomers

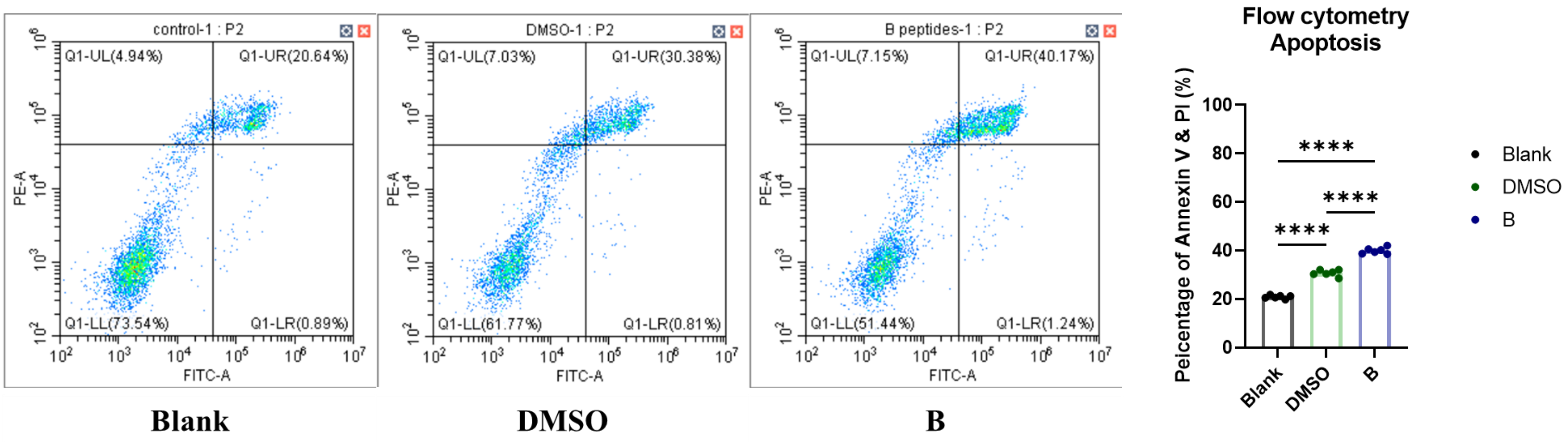

Figure S9. Functional assay of apoptosis

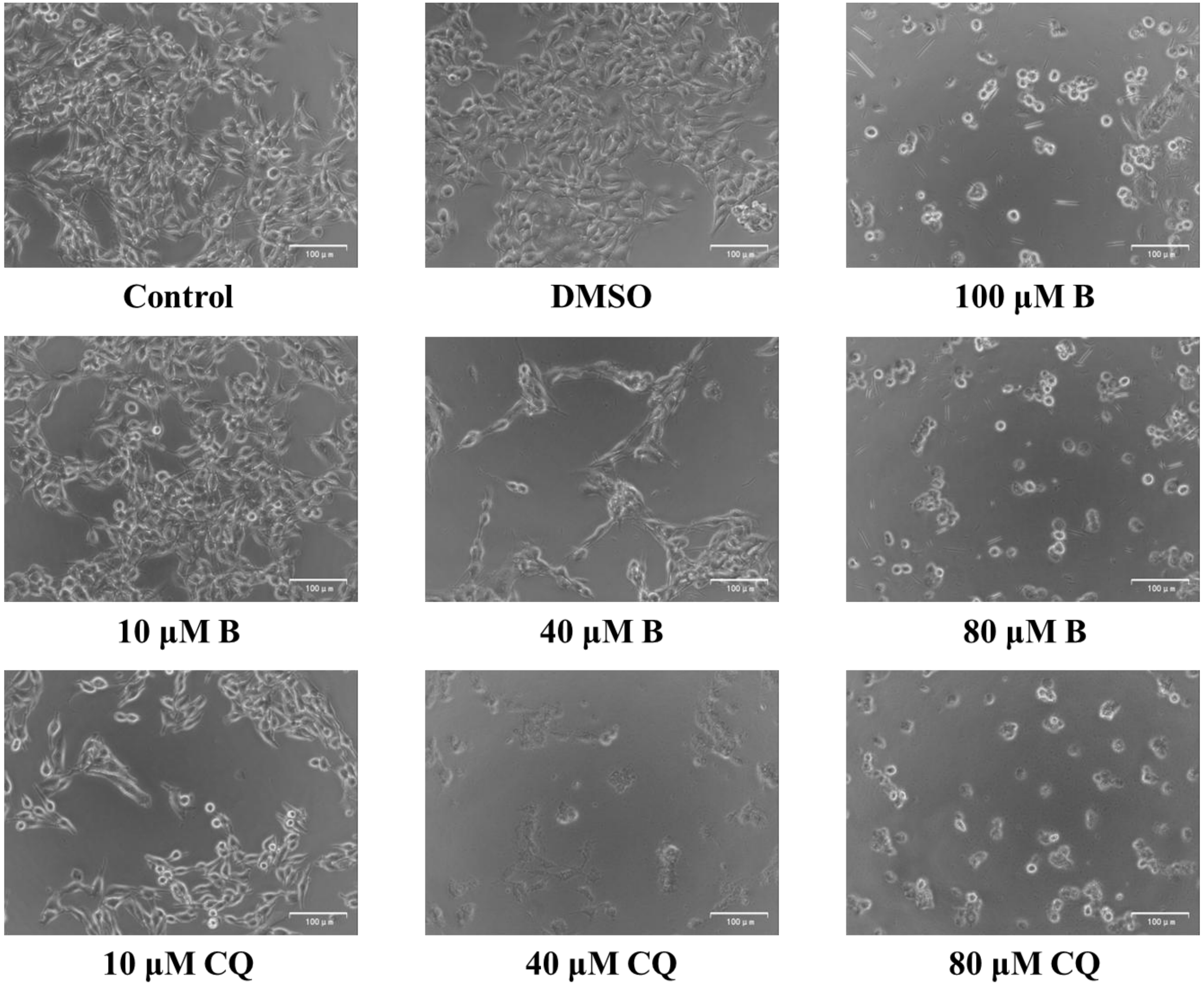

Figure S10. Phase contrast microscope photos of B16-F10 after 24 hours

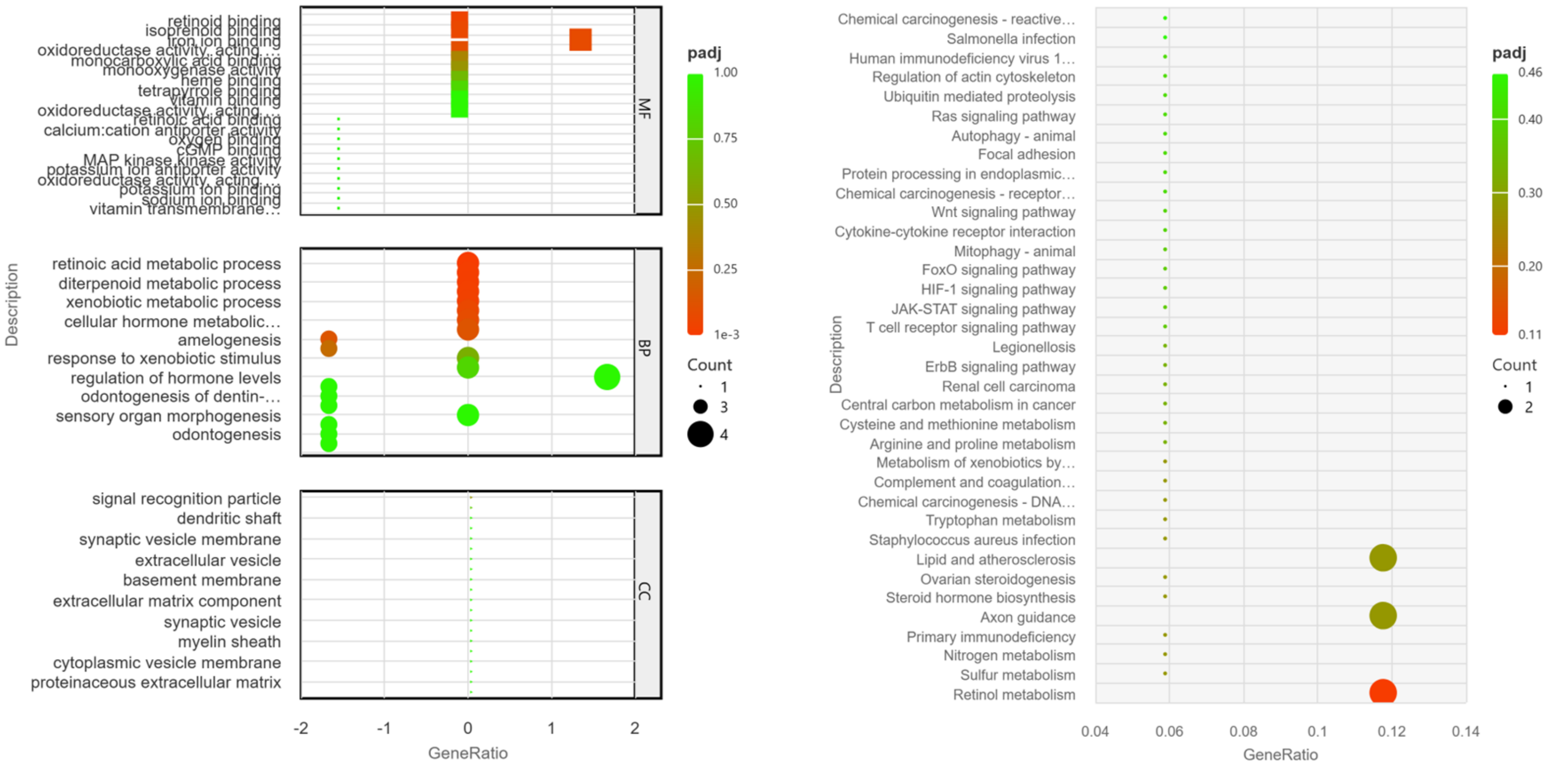

Figure S11. RNAseq: KEGG and GO

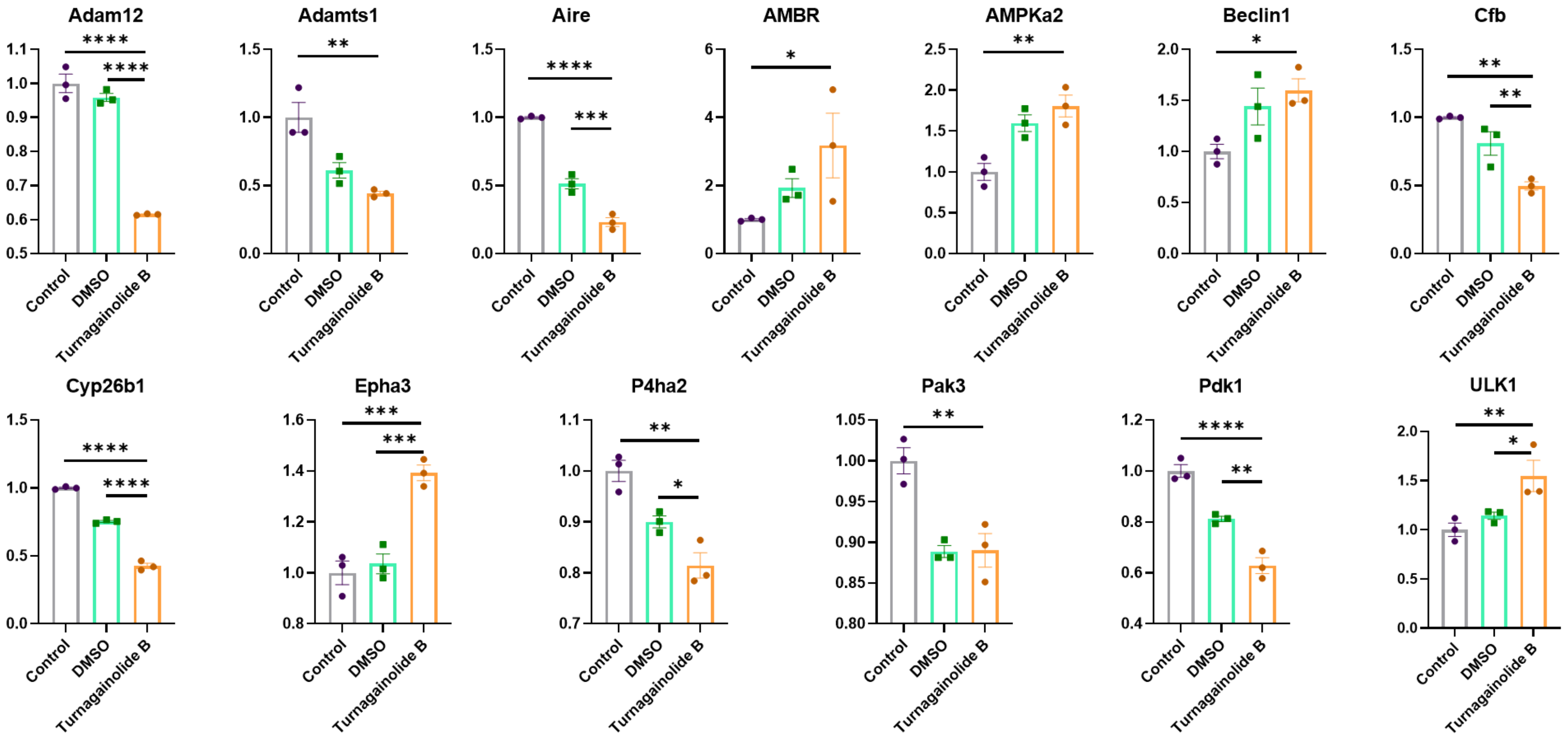

Figure S12. qPCR results related to cell migration, tumor immunology and autophagy (2 ~ 4 hours)

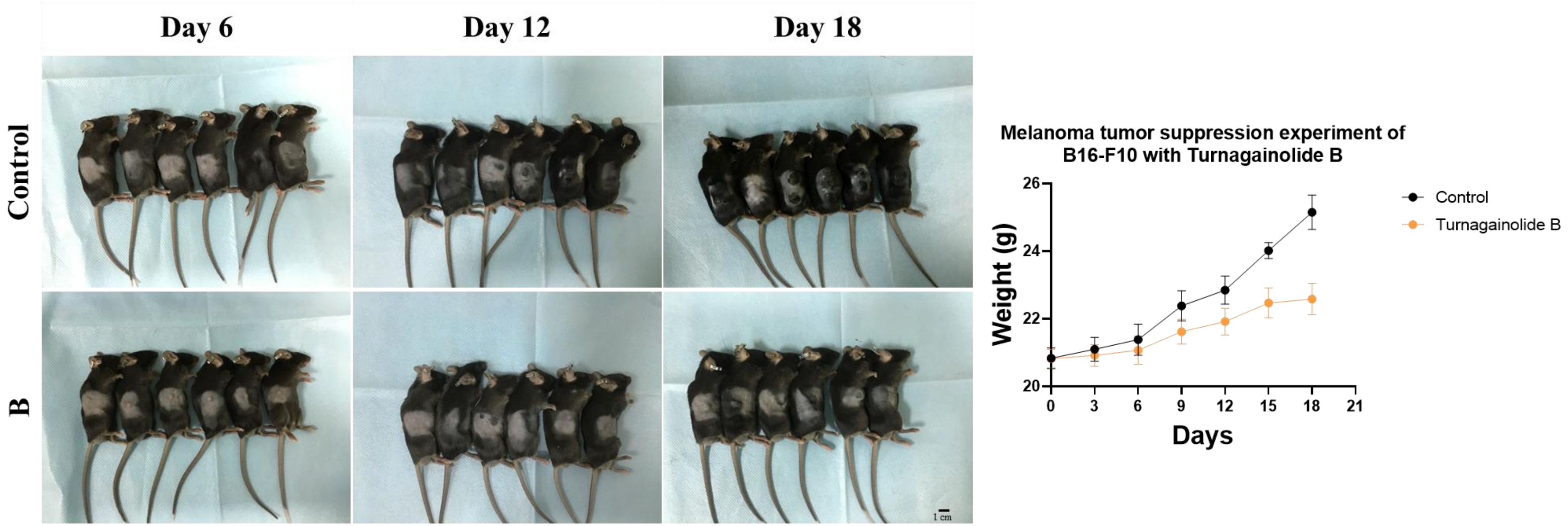

Figure S13. Animal experiment: mouse photo and weight

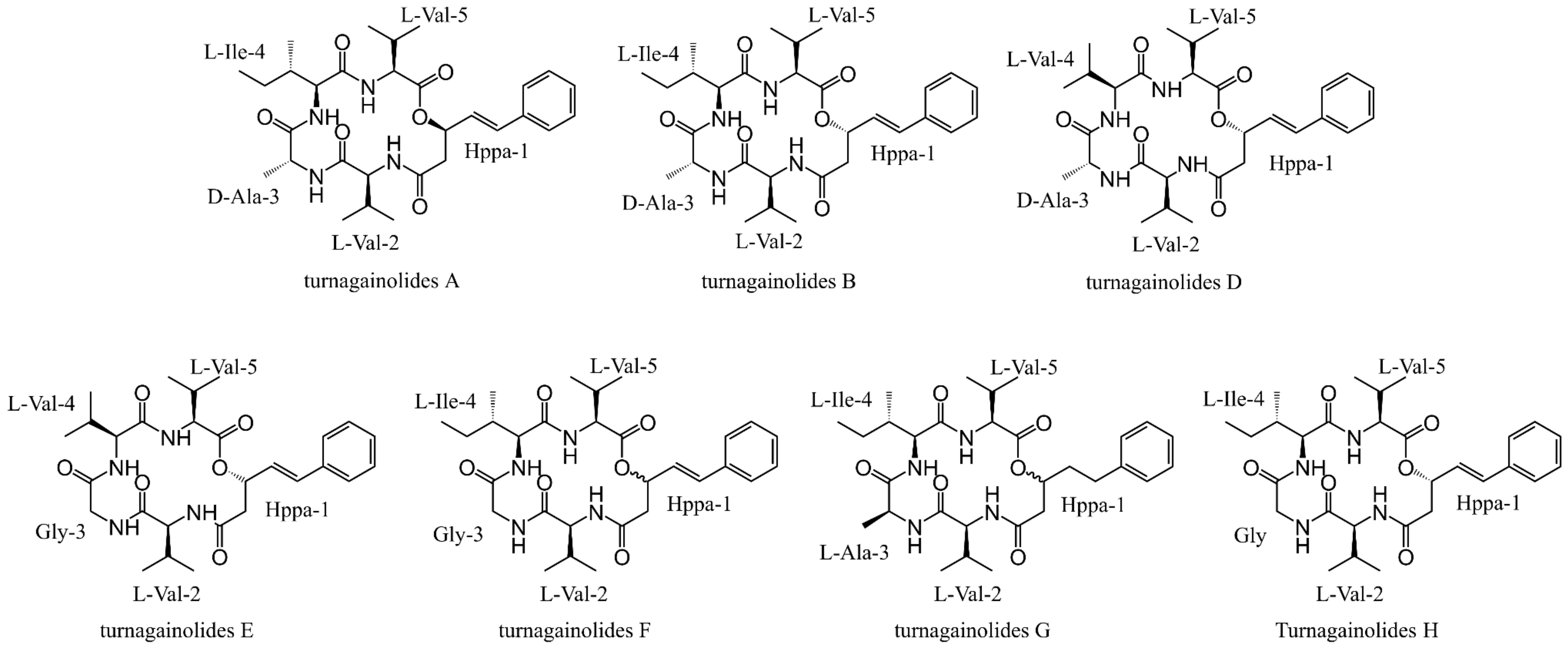

Figure S14. The chemical structures of turnagainolides family

Supporting Table S1

SwissTargetPrediction						
**Target**	**Common name**	**Uniprot ID**	**ChEMBL ID**	**Target Class**	**Probability***	**Known actives (3D/2D)**
Histone deacetylase 6 (by homology)	Hdac6	Q9Z2V5	CHEMBL2878	Eraser	0	162 / 22
Histone deacetylase 1 (by homology)	Hdac1	O09106	CHEMBL4001	Eraser	0	438 / 28
Cholecystokinin A receptor	Cckar	O08786	CHEMBL2798	Family A G protein-coupled receptor	0	94 / 0
cAMP and cAMP-inhibited cGMP 3',5'-cyclic phosphodiesterase 10A (by homology)	Pde10a	Q8CA95	CHEMBL1795126	Phosphodiesterase	0	1287 / 0
Dopamine D1 receptor (by homology)	Drd1	Q61616	CHEMBL3071	Family A G protein-coupled receptor	0	38 / 0
Acyl coenzyme A:cholesterol acyltransferase 1 (by homology)	Soat1	Q61263	CHEMBL4464	Enzyme	0	32 / 6
Peroxisome proliferator-activated receptor alpha (by homology)	Ppara	P23204	CHEMBL2128	Nuclear receptor	0	9 / 0
11-beta-hydroxysteroid dehydrogenase 1 (by homology)	Hsd11b1	P50172	CHEMBL3910	Enzyme	0	410 / 0
Orexin receptor type 2 (by homology)	Hcrtr2	P58308	CHEMBL2434818	Family A G protein-coupled receptor	0	1079 / 0
Orexin receptor type 1 (by homology)	Hcrtr1	P58307	CHEMBL2434819	Family A G protein-coupled receptor	0	935 / 0
Serotonin 2c (5-HT2c) receptor (by homology)	Htr2c	P34968	CHEMBL3006	Family A G protein-coupled receptor	0	225 / 0
Glucokinase (by homology)	Gck	P52792	CHEMBL3112387	Enzyme	0	154 / 0
MAP kinase p38 alpha (by homology)	Mapk14	P47811	CHEMBL2336	Kinase	0	1008 / 0
Macrophage-stimulating protein receptor (by homology)	Mst1r	Q62190	CHEMBL1795170	Kinase	0	16 / 0
Peripheral-type benzodiazepine receptor (by homology)	Tspo	P50637	CHEMBL2149	Membrane receptor	0	310 / 0
E3 ubiquitin-protein ligase Mdm2 (by homology)	Mdm2	P23804	CHEMBL3600279	Ligase	0	256 / 0
Hypoxia-inducible factor 1-alpha (by homology)	Hif1a	Q61221	CHEMBL6046	Transcription factor	0	14 / 0
Mitogen-activated protein kinase 9 (by homology)	Mapk9	Q9WTU6	CHEMBL2034797	Kinase	0	151 / 0
Insulin receptor (by homology)	Insr	P15208	CHEMBL3187	Kinase	0	84 / 0
Ribosomal protein S6 kinase alpha-3 (by homology)	Rps6ka3	P18654	CHEMBL3297641	Enzyme	0	117 / 0
Tyrosine-protein kinase JAK3 (by homology)	Jak3	Q62137	CHEMBL5250	Kinase	0	278 / 0
Serine/threonine-protein kinase 12 (by homology)	Aurkb	O70126	CHEMBL1075275	Kinase	0	287 / 0
Bromodomain-containing protein 9 (by homology)	Brd9	Q3UQU0	CHEMBL3822347	Reader	0	8 / 0
Serine/threonine-protein kinase Aurora-A (by homology)	Aurka	P97477	CHEMBL2211	Kinase	0	483 / 0
Acetyl-CoA carboxylase 2 (by homology)	Acacb	E9Q4Z2	CHEMBL3108631	Ligase	0	278 / 0
Sodium channel protein type 9 subunit alpha (by homology)	Scn9a	Q62205	CHEMBL3414411	Voltage-gated ion channel	0	185 / 0
Voltage-gated potassium channel subunit Kv1.3 (by homology)	Kcna3	P16390	CHEMBL4818	Voltage-gated ion channel	0	53 / 0
Tankyrase-1 (by homology)	Tnks	Q6PFX9	CHEMBL3232702	Enzyme	0	72 / 0
Neuropeptide Y receptor type 5 (by homology)	Npy5r	O70342	CHEMBL3802	Family A G protein-coupled receptor	0	364 / 0
Adenosylhomocysteinase (by homology)	Ahcy	P50247	CHEMBL2389	Enzyme	0	8 / 0
Scavenger receptor class B member 1 (by homology)	Scarb1	Q61009	CHEMBL1741203	Unclassified protein	0	41 / 0
Cathepsin S	Ctss	O70370	CHEMBL4098	Protease	0	4 / 0
Probable protein-cysteine N-palmitoyltransferase porcupine	Porcn	Q9JJJ7	CHEMBL1255164	Enzyme	0	53 / 0
Arachidonate 5-lipoxygenase (by homology)	Alox5	P48999	CHEMBL5211	Oxidoreductase	0	123 / 0
Protein kinase C delta (by homology)	Prkcd	P28867	CHEMBL2560	Kinase	0	37 / 0
Protein kinase C theta type (by homology)	Prkcq	Q02111	CHEMBL1075295	Kinase	0	49 / 0
Prostanoid EP1 receptor (by homology)	Ptger1	P35375	CHEMBL2181	Family A G protein-coupled receptor	0	78 / 0
Serotonin 4 (5-HT4) receptor (by homology)	Htr4	P97288	CHEMBL2183	Family A G protein-coupled receptor	0	19 / 0
Adenosine A2b receptor (by homology)	Adora2b	Q60614	CHEMBL2237	Family A G protein-coupled receptor	0	134 / 0
Fatty acid synthase (by homology)	Fasn	P19096	CHEMBL1795189	Transferase	0	152 / 0
Phosphodiesterase 4B (by homology)	Pde4b	Q8VBU5	CHEMBL2272	Phosphodiesterase	0	273 / 0
Protein-tyrosine kinase 2-beta	Ptk2b	Q9QVP9	CHEMBL1075289	Kinase	0	6 / 0
Epidermal growth factor receptor erbB1 (by homology)	Egfr	Q01279	CHEMBL3608	Kinase	0	610 / 0
Adenosine A1 receptor (by homology)	Adora1	Q60612	CHEMBL3688	Family A G protein-coupled receptor	0	656 / 0
Adenosine A2a receptor (by homology)	Adora2a	Q60613	CHEMBL2115	Family A G protein-coupled receptor	0	554 / 0
TGF-beta receptor type-1 (by homology)	Tgfbr1	Q64729	CHEMBL2021750	Kinase	0	214 / 0
Vascular endothelial growth factor receptor 1 (by homology)	Flt1	P35969	CHEMBL3516	Kinase	0	150 / 0
MAP kinase-activated protein kinase 2 (by homology)	Mapkapk2	P49138	CHEMBL4990	Kinase	0	118 / 0
Mu opioid receptor (by homology)	Oprm1	P42866	CHEMBL2858	Family A G protein-coupled receptor	0	81 / 93

Target	Common name	Uniprot ID	ChEMBL ID	Target Class	Probability*	Known actives (3D/2D)
Delta opioid receptor	Oprd1	P32300	CHEMBL3222	Family A G protein-coupled receptor	0	74 / 92
Glucose-dependent insulinotropic receptor (by homology)	Gpr119	Q7TQP3	CHEMBL5263	Family A G protein-coupled receptor	0	48 / 0
Liver glycogen phosphorylase (by homology)	Pygl	Q9ET01	CHEMBL3008	Enzyme	0	54 / 0
High affinity cAMP-specific 3',5'-cyclic phosphodiesterase 7A (by homology)	Pde7a	P70453	CHEMBL2040702	Phosphodiesterase	0	130 / 0
Serine/threonine-protein kinase B-raf (by homology)	Braf	P28028	CHEMBL2331061	Kinase	0	206 / 0
Phosphatidylinositol 4,5-bisphosphate 3-kinase catalytic subunit gamma isoform (by homology)	Pik3cg	Q9JHG7	CHEMBL2189158	Enzyme	0	212 / 0
PI3-kinase p110-alpha subunit (by homology)	Pik3ca	P42337	CHEMBL2499	Enzyme	0	596 / 0
Sodium- and chloride-dependent glycine transporter 1 (by homology)	Slc6a9	P28571	CHEMBL1075303	Electrochemical transporter	0	147 / 0
DNA-dependent protein kinase catalytic subunit (by homology)	Prkdc	P97313	CHEMBL2176779	Kinase	0	54 / 0
Nuclear receptor ROR-gamma (by homology)	Rorc	P51450	CHEMBL1293231	Nuclear receptor	0	136 / 0
Nitric oxide synthase, inducible (by homology)	Nos2	P29477	CHEMBL3464	Enzyme	0	100 / 0
Histamine H1 receptor (by homology)	Hrh1	P70174	CHEMBL4322	Family A G protein-coupled receptor	0	36 / 0
Glycogen synthase kinase-3 beta (by homology)	Gsk3b	Q9WV60	CHEMBL1075321	Kinase	0	371 / 0
Ectonucleotide pyrophosphatase/phosphodiesterase family member 2 (by homology)	Enpp2	Q9R1E6	CHEMBL3826871	Enzyme	0	8 / 0
ALK tyrosine kinase receptor (by homology)	Alk	P97793	CHEMBL5771	Kinase	0	113 / 0
Mast/stem cell growth factor receptor Kit (by homology)	Kit	P05532	CHEMBL2034798	Kinase	0	128 / 0
Thyrotropin-releasing hormone receptor	Trhr	P21761	CHEMBL2467	Family A G protein-coupled receptor	0	34 / 0
Tyrosine-protein kinase ABL (by homology)	Abl1	P00520	CHEMBL3099	Kinase	0	195 / 0
Thyrotropin-releasing hormone receptor 2	TRH-R2	Q9ERT1	CHEMBL3600274	Family A G protein-coupled receptor	0	28 / 0
Platelet activating factor receptor (by homology)	Ptafr	Q62035	CHEMBL3993	Family A G protein-coupled receptor	0	74 / 0
Nicotinamide phosphoribosyltransferase (by homology)	Nampt	Q99KQ4	CHEMBL3259474	Enzyme	0	155 / 0
Neuronal acetylcholine receptor protein alpha-7 subunit (by homology)	Chrna7	P49582	CHEMBL3365	Ligand-gated ion channel	0	23 / 0
Tyrosine-protein kinase BTK (by homology)	Btk	P35991	CHEMBL3259478	Kinase	0	51 / 0
Proto-oncogene tyrosine-protein kinase receptor Ret (by homology)	Ret	P35546	CHEMBL2034799	Kinase	0	52 / 0
Elongation of very long chain fatty acids protein 6 (by homology)	Elovl6	Q920L5	CHEMBL5726	Enzyme	0	23 / 0
MAP kinase p38 beta	Mapk11	Q9WUI1	CHEMBL4335	Kinase	0	54 / 0
Legumain (by homology)	Lgmn	O89017	CHEMBL1949492	Protease	0	55 / 0
Serine/threonine-protein kinase TBK1 (by homology)	Tbk1	Q9WUN2	CHEMBL2189160	Kinase	0	33 / 0
Cyclooxygenase-1 (by homology)	Ptgs1	P22437	CHEMBL2649	Enzyme	0	47 / 0
Indoleamine 2,3-dioxygenase 1 (by homology)	Ido1	P28776	CHEMBL1075294	Enzyme	0	38 / 0
Alpha-1,6-mannosyl-glycoprotein 2-beta-N-acetylglucosaminyltransferase (by homology)	Mgat2	Q921V5	CHEMBL2375203	Enzyme	0	37 / 0
C-X-C chemokine receptor type 3 (by homology)	Cxcr3	O88410	CHEMBL5200	Family A G protein-coupled receptor	0	44 / 0
Histone deacetylase 8 (by homology)	Hdac8	Q8VH37	CHEMBL2347	Eraser	0	55 / 7
Tankyrase-2 (by homology)	Tnks2	Q3UES3	CHEMBL3232703	Enzyme	0	49 / 0
Acyl-CoA desaturase 1	Scd1	P13516	CHEMBL5353	Enzyme	0	103 / 0
RAF proto-oncogene serine/threonine-protein kinase (by homology)	Raf1	Q99N57	CHEMBL3804748	Kinase	0	103 / 0
Multidrug resistance-associated protein 1 (by homology)	Abcc1	O35379	CHEMBL2532	Primary active transporter	0	19 / 0
Metabotropic glutamate receptor 5 (by homology)	Grm5	Q3UVX5	CHEMBL1641352	Family C G protein-coupled receptor	0	688 / 0
Dual-specificity tyrosine-phosphorylation regulated kinase 1A (by homology)	Dyrk1a	Q61214	CHEMBL4750	Kinase	0	109 / 0
Estrogen receptor alpha (by homology)	Esr1	P19785	CHEMBL3065	Nuclear receptor	0	43 / 0
Dual specificity protein kinase CLK1 (by homology)	Clk1	P22518	CHEMBL1075280	Enzyme	0	51 / 0
Dual specificity protein kinase CLK2 (by homology)	Clk2	O35491	CHEMBL1075281	Enzyme	0	16 / 0
Protein Wnt-3a (by homology)	Wnt3a	P27467	CHEMBL5617	Unclassified protein	0	7 / 0
Inosine-5'-monophosphate dehydrogenase 2 (by homology)	Impdh2	P24547	CHEMBL3169	Oxidoreductase	0	77 / 0
Protein kinase C eta (by homology)	Prkch	P23298	CHEMBL4992	Kinase	0	4 / 0
Bcl-2-related protein A1	Bcl2a1	Q07440	CHEMBL1293239	Unclassified protein	0	15 / 0
Receptor-interacting serine/threonine-protein kinase 1 (by homology)	Ripk1	Q60855	CHEMBL3784911	Kinase	0	16 / 0
Macrophage colony-stimulating factor 1 receptor (by homology)	Csf1r	P09581	CHEMBL5570	Kinase	0	198 / 0
Plasminogen (by homology)	Plg	P20918	CHEMBL1075299	Protease	0	1 / 0
Monoamine oxidase A (by homology)	Maoa	Q64133	CHEMBL3681	Enzyme	0	122 / 0
Dual specificity mitogen-activated protein kinase kinase 1 (by homology)	Map2k1	P31938	CHEMBL5860	Enzyme	0	90 / 0
